# High-glucose diets differentially modulate phosphatidylcholine metabolism and fecundity in *Caenorhabditis elegans*

**DOI:** 10.3389/fcell.2025.1622695

**Published:** 2025-08-29

**Authors:** Chao-Wen Wang, Phebe Chiu, Sophia Monsalve, Ricardo Roure, Xiaofei Bai, Jia-Jin Law, Yu-Ching Wu, Yet-Ran Chen, You-Liang Cheng, Rey-Huei Chen, Yi-Chun Wu

**Affiliations:** ^1^ Department of Life Sciences, National Cheng Kung University, Tainan, Taiwan; ^2^ Institute of Plant and Microbial Biology, Academia Sinica, Taipei, Taiwan; ^3^ Department of Biology and Genetics Institute, University of Florida, Gainesville, FL, United States; ^4^ Institute of Agricultural Biotechnology Research Center, Academia Sinica, Taipei, Taiwan; ^5^ Institute of Molecular Biology, Academia Sinica, Taipei, Taiwan; ^6^ Institute of Molecular and Cellular Biology, National Taiwan University, Taipei, Taiwan

**Keywords:** glucose, diet, phosphatidylcholine, triacylglycerol, choline, methionine, vitamin B12, omics

## Abstract

**Background:**

*Caenorhabditis elegans* fed a high-glucose Escherichia coli OP50 diet exhibit reduced fecundity, but the underlying mechanisms remain unclear.

**Methods:**

A differential high-glucose diet paradigm was established using *C. elegans* fed two bacterial diets that produced distinct fecundity outcomes under high-glucose conditions. The effects of these diets in varying conditions were analyzed through transcriptomic, lipidomic, and metabolomic profiling to correlate with fecundity. Supplementation experiments were further performed to validate the links between changes in lipid metabolism and fecundity. By characterizing the gerlime phenotypes, we constructed a model to interpret how dietary inputs alter oogenesis signaling and, consequently, fecundity outcomes.

**Results:**

*C. elegans* fed a high-glucose *E. coli* OP50 diet exhibit reduced fecundity, accompanied by disrupted lipid homeostasis characterized by decreased monounsaturated and increased cyclopropane fatty acids, reduced phosphatidylcholine and elevated triacylglycerols, and abnormal lipid droplet and vitellogenin accumulation in the intestine and oocytes. In contrast, worms fed a high-glucose *Comamonas aquatica* DA1877 diet maintain lipid balance and normal fecundity. We identified altered lipid metabolism strongly correlated with reproductive decline, whereas dietary signals from *C. aquatica* protected against glucose toxicity. Mechanistically, high-glucose diets appeared to rewire the choline–methionine axis, lowering PC levels and reducing RAS/ERK signaling in germline and gonadal sheath cells, thereby impairing oogenesis. Notably, vitamin B12 supplementation restored RAS/ERK signaling and rescued the diet-specific fecundity defects.

**Conclusion:**

We demonstrate that dietary cues under high-glucose conditions modulate a genetic network linking lipid homeostasis and signaling pathways, ultimately determining fecundity outcomes in *C. elegans*.

## Introduction

Glucose is a fundamental energy source and metabolic precursor in most organisms. Beyond providing energy, glucose supplies carbon for the synthesis of numerous biomolecules, including nucleotides, amino acids, lipids, and structural polysaccharides. Excess glucose is stored as glycogen for short-term energy needs and as triacylglycerol (TAG) for long-term storage. However, the consumption of high-sugar diets could be detrimental to humans, such as by causing obesity, a known risk factor for various diseases, including type 2 diabetes (Chatterjee et al., 2017).

Glucose-induced deleterious effects have been identified in *C. elegans*, with well-documented impacts on growth, fertility, aging, and lifespan (Back et al., 2012; Beaudoin-Chabot et al., 2022; Schlotterer et al., 2009). The lifespan-shortening effect of high glucose in *C. elegans* has been extensively studied, which is partly attributed to the activation of the insulin/insulin-like growth factor-1 signaling (IIS) pathway (Lee et al., 2009; Schlotterer et al., 2009). High glucose levels generate reactive oxygen species (ROS) that can damage lipids, proteins, and nucleic acids (Volpe et al., 2018). *C. elegans* fed a high-glucose diet exhibit an increased rate of lipid peroxidation, indicating elevated oxidative stress (Alcantar-Fernandez et al., 2019; Alcantar-Fernandez et al., 2018). However, increased ROS levels extend the lifespan of worms, suggesting that a certain level of ROS appears essential for cellular function and longevity (Lee et al., 2010; Schmeisser et al., 2013; Schulz et al., 2007; Shore and Ruvkun, 2013; Yang and Hekimi, 2010).

At the cellular level, surplus glucose alters mitochondrial structure and function, yet energy homeostasis is maintained (Alcantar-Fernandez et al., 2019), indicating that high glucose’s harmful effects stem from more than just ATP shortage or oxidative stress. High glucose also leads to glycogen and fat accumulation, with SREBP and MDT-15 regulating fat conversion to mitigate high-glucose diet-induced aging (Lee et al., 2015). Although glycogen confers resistance to oxidative stress, it limits longevity (Gusarov et al., 2017). Given the multifaceted physiological effects of high glucose, elucidating the exact mechanisms by which surplus glucose causes diverse effects is essential, despite the significant challenges involved.

Diet represents one of the most variable aspects of life (Fontana and Partridge, 2015). Diet not only provides nutrients but also influences the intestinal microbiome, which, along with microbiome-derived metabolites, impacts overall animal metabolism and immune responses (Hooper et al., 2002). As a bacterivore, *C. elegans* provides a powerful system that can be maintained on monocultures under laboratory conditions, enabling mechanistic studies on various physiological aspects (Pang and Curran, 2014; Stuhr and Curran, 2020). Studies have revealed that *C. elegans* responds differently to bacterial diets: *Comamonas aquatica* DA1877 and *Escherichia coli* OP50. Vitamin B12, abundant in the *C. aquatica* DA1877 diet, is essential for *C. elegans* metabolism, supporting both mitochondrial propionate breakdown and one-carbon metabolism (Giese et al., 2020; MacNeil et al., 2013; Watson et al., 2013; Watson et al., 2014; Watson et al., 2016). Accordingly, *C. elegans* fed *C. aquatica* DA1877 exhibit phenotypes such as accelerated developmental rates and earlier onset of egg laying compared to those fed *E. coli* OP50. In contrast, low vitamin B12 availability leads to toxic propionate accumulation, which triggers the expression of several genes, including the *acyl-CoA dehydrogenase* genes *acdh-1* and *acdh-2*. It also represses the expression of genes involved in methionine/S-adenosylmethionine (SAM) metabolism, a key component of one-carbon metabolism. This route produces the main methyl donor in the cell, which is pivotal to many critical cellular processes, including nucleotide and protein methylation and the synthesis of membrane lipids (Giese et al., 2020; Laranjeira et al., 2024; Sutter et al., 2013).

In this study, we examine how high-glucose intake reduces fecundity in *C. elegans* fed the standard *E. coli* OP50 diet. We first observed that *C. elegans* fed *C. aquatica* DA1877 diets did not experience the same effect under high-glucose conditions. We hypothesize that differing bacterial diets trigger distinct metabolic pathways in response to glucose, leading to unique metabolic and gene regulatory outcomes. Through transcriptomic and lipidomic analyses and comparison, we have identified that the differential effect of a high-glucose diet is associated with a change in fatty acid (FA), phosphatidylcholine (PC), and TAG levels, affecting lipid homeostasis. We further reveal that the metabolic pathway involving the choline–methionine/SAM axis responds to dietary signals under high-glucose conditions, potentially influencing organismal fecundity through varying PC levels and oogenesis signaling.

## Materials and methods

### *C. elegans* strains, culture, and maintenance

A list of bacterial and *C. elegan*s strains, as well as the reagents and materials used in this study, is available in the Table 1. The *C. elegans* strain Bristol N2 was used as the wild type. *C. elegans* were maintained on standard nematode growth medium (NGM) plates seeded with *E. coli* OP50 and were cultured at 20 °C. *E. coli* OP50 and *C. aquatica* DA1877 were grown overnight at 37 °C and 30 °C, respectively, in LB liquid medium. The bacterial cells were spun down and washed in ddH_2_O, and A_600_ was adjusted to 8.0. The NGM or NGM containing 100 mM glucose (high glucose, HG) plates were seeded with *E. coli* OP50, *C. aquatica* DA 1877, *E. coli* OP50: *C. aquatica* DA 3:1, or the *E. coli* OP50: *C. aquatica* DA 9:1 prior to the experiment. Both 50 mM and 100 mM used in this study are supraphysiological levels of glucose that lack clinical or ecological relevance but serve as useful experimental conditions for studying metabolic perturbations. The plates were dried at room temperature for 1–1.5 days, and only freshly prepared plates were used in all experiments. Synchronized L1 animals were used for experiments unless otherwise mentioned. All experiments were conducted at 20 °C.

**TABLE 1:** Key resource table.

Reagent or resource	Source	Identifier
Bacteria strain		
*Escherichia coli* OP50	*Caenorhabditis* Genetics Center (CGC)	OP50
*Comamonas aquatica* DA1877	CGC	DA1877
*C. elegans* strains		
N2 Bristol	CGC	N2
*nIs590[fat-7p::fat-7::gfp + lin15(+)]V*	CGC	DMS303
*ldrIs1 [Pdhs-3::dhs-3::gfp + unc-76(+)]*	CGC	LIU1
*pwls23 [vit-2::gfp]*	CGC	RT130
*ruls32 [pie-1p::GFP::H2B + unc-119(+)] III*	CGC	MAH74
*unc-119(ed3) III; wwEx53[acdh-2p::GFP]*	CGC	VL714
*mpk-1(utx14[mNG::mpk-1]) III*	CGC	GLW19
*let-60(qy220[mNG::let-60 + LoxP]) IV*	CGC	NK2987
wrdSi18[^SEC^mex-5p::TIR1::F2A::mTagBFP2::AID*::NLS::tbb-2 3′UTR] (I:-5.32)	CGC	JDW220
Chemical, peptide, and recombinant protein		
TRIzol reagent	Sigma	T9424
Phenol: chloroform: isoamyl alcohol 25: 24: 1	AppliChem	A2279
RNeasy Mini Kits	QIAGEN	74104
Low Input Quick-Amp Labeling Kit	Agilent Technologies	
Levamisole hydrochloride	Sigma	L9756
Agarose	BioShop	AGA101.25
Chloroform	J.T.Baker	9183–01
Methanol	J.T.Baker	9069–01
Boron trifluoride–methanol 10%–15%	Sigma-Aldrich	15716
Tridecanoic acid	Sigma	91988
Choline chloride	Sigma	C7527
Methionine	Sigma	M9625
Palmitic acid	Sigma	P0500
Oleic acid	Sigma	O1383
cis-Vaccenic acid	Sigma	V0384
α-Linolenic acid	Sigma	L2376
Brij58	Sigma	P5884
Betaine	Sigma	107437
Cobalamin (vitamin B12)	Sigma	C0884
Sodium hydroxide	J.T.Baker	3140–01
Anti-GFP antibody	Abcam	ab290
Oligonucleotide		
5’_QP_act-1 primer CTTCCCTCTCCACCTTCCAAC	This paper	
3’_QP_act-1 primer CGTCGTATTCTTGCTTGGAGATC	This paper	
5’_QP_acs-2 primer GATGCTCATGTCGTCGGTGTG	This paper	
3’_QP_acs-2 primer CGACAGCTTCTGTGAAGTGAG	This paper	
5’_QP_fat-5 primer ACAGTTGGATGGGTATTCCTC	This paper	
3’_QP_fat-5 primer AAGCAGAAGATTCCGACCAAG	This paper	
5’_QP_fat-6 primer AACATTACTTCCCACTTGTC	This paper	
3’_QP_fat-6 primer GGGTAATTGAGGAATCGTATG	This paper	
5’_QP_fat-7 primer CGTCTTCTCATTTGCTCTCTATGTG	This paper	
3’_QP_fat-7 primer TGACCAGTGGGAAATAGTGC	This paper	
5’_QP_arf-1.1 primer TTCGTGCTCTCTGGAAATAC	This paper	
3’_QP_arf-1.1 primer TGTACCACAGATGAACCACTC	This paper	
5’_QP_asm-3 primer CTCCAACTAATGTGGTCTACTCG	This paper	
3’_QP_asm-3 primer GTCGGGATTTGTCCTTTAAGACC	This paper	
5’_QP_pud-4 primer ACACTGGATTCTTCACGACTGG	This paper	
3’_QP_pud4 primer TCCATTTGGCGAGTAGTCCATG	This paper	
5’_QP_lipl-2 primer ACAGTCACTACGGAAGATGG	This paper	
3’_QP_lipl-2 primer CGAAAGCTGCACTCTGAGTTG	This paper	
5’_QP_lipl-1 primer GACCACGCCACAAATAATCATGC	This paper	
3’_QP_lipl-1 primer TGATGAGCATTCAAGACCGTG	This paper	
5’_QP_fil-1 primer CGTCGATTCGTACTTCTTGGTC	This paper	
3’_QP_fil-1 primer AGCTTCCCTGAGTTCCATAGTC	This paper	
5’_QP_sysm-1 primer AGCAGATGTTAGAAGTGGTTGTC	This paper	
3’_QP_sysm-1 primer CAGATTTCTGCATTGGCTGTG	This paper	
5’_QP_gba-4 primer AGCGATGCAAAGTCCCTTCC	This paper	
3’_QP_gba-4 primer GCAGCTCCAAATCCCATTACC	This paper	
5’_QP_dhs-26 primer CAGTGACTAAATGCGGATCAGG	This paper	
3’_QP_dhs-26 primer TTTCCAACACCATAGGCGACG	This paper	
5’_QP_acdh-2_ACE primer GGAGACCACTTCATTCTCAACG	This paper	
3’_QP_ acdh-2_ACE primer TGAGCAATGGTTCCTGCACGC	This paper	
Software and algorithms		
ImageJ	Schneider et al.	https://imagej.nih.gov/ij/
GraphPad Prism	Dotmatics	https://www.dotmatics.com/

### mRNA isolation and microarray analysis

*C. elegans* eggs were isolated from gravid adults using hypochlorite disruption (0.5 N NaOH and 1% NaOCl), followed by washing with M9 buffer. After overnight incubation in M9 devoid of food, synchronized L1 were seeded on 5.5-cm NGM plates (∼2000 L1/plate for a total of ∼10000 L1/diet treatment). RNAs were isolated from the young adult animals, defined at the onset of egg laying, just after the L4-to-adult transition, with visible eggs in the uterus (50 h for DA, 52 h for HG-DA, 56 h for OP, and 62 h for HG-OP post-L1 feeding). The animals were harvested, washed with M9 buffer, and pelleted. TRIzol reagent (500 μL per 100 μL worm pellet) was added, followed by flash-freezing in liquid nitrogen. The samples of TRIzol reagent/worm pellets were thawed at 42 °C for 3 min, vortexed for 30 s, and frozen in liquid nitrogen again for 3 min. This cycle was repeated seven times. To extract total RNA, 100 μL of phenol: chloroform: isoamyl alcohol 25: 24: 1 (AppliChem, Darmstadt, Germany) was mixed with the sample, incubated at room temperature for 3 min, and spun at 12,000 g for 15 min at 4 °C. The aqueous layer was isolated and transferred to an RNeasy spin column of the RNeasy Mini Kits (QIAGEN, Hilden, Germany) for further purification, following the manufacturer’s instructions. RNA concentration was determined using a NanoDrop spectrophotometer.

To prepare samples for microarray analysis, 0.2 μg of total RNA was subjected to Cy3 labeling using the Low Input Quick-Amp Labeling Kit (Agilent Technologies, United States) to prepare Cy3-labeled cRNA. After incubation at 60 °C for 30 min, 1.65 μg of Cy3-labeled cRNA was hybridized to the Agilent *C. elegans* V2 4*44K Microarray Chip (Agilent Technologies, United States) at 65 °C for 17 h; following washing and drying steps, the microarray chip was scanned using a microarray scanner (Agilent Technologies, United States) at 535 nm for Cy3. The microarray image was analyzed using Feature Extraction software version 10.7.1.1 with the default settings. These data were then subjected to further analysis in Microsoft Excel by transforming the values to ratios (fold change, FC) corresponding to HG-OP/OP and HG-DA/DA, and three independent replicates were averaged. The genes for HG-OP/OP that had average Log_2_ FC values < −1 or >1 and p-values <0.05 were selected and sorted to prepare the gene expression heatmap shown in Figure 2A. Genes that passed these criteria were subjected to functional enrichment using g:GOSt in g:Profiler (https://biit.cs.ut.ee/gprofiler/gost) against the KEGG pathway (Raudvere et al., 2019). Raw data from three independent microarray experiments were analyzed using principal component analysis in ClustVis (https://biit.cs.ut.ee/clustvis/).

### Quantitative PCR

*C. elegans* were synchronized using hypochlorite disruption as described. The synchronized young adults (50 h for DA, 52 h for HG-DA, 55 h for mix 9:1, 56 h for OP, 56 h for mix 3:1, and 62 h for HG-OP post L1 feeding) were harvested, washed with M9 buffer, and pelleted (∼2000 young adults per diet treatment). RNA was isolated using TRIzol and RNeasy Mini Kits (QIAGEN) as described, followed by treatment with DNase I (QIAGEN), to obtain pure RNA. RNA (8 μg) was used as the template for cDNA synthesis using the SuperScript III First-Strand System (Thermo Fisher Scientific). The qPCR reactions were completed using the Applied Biosystems QuantStudio 12K Flex System with the Power SYBR Green PCR Master Mix (Thermo Fisher Scientific, Waltham, MA, United States). The thermal cycling conditions began with a hold stage at 50 °C for 2 min, followed by 95 °C for 10 min, and then 40 cycles of 95 °C for 15 s and 60 °C for 1 min The ΔCT value was processed using QuantStudio 12K Flex software (Thermo Fisher Scientific). Gene expression levels were calculated as 2^(−ΔΔCT)^ and normalized to OP samples and *act-1* gene expression. Three to four independent experiments were averaged for each target gene (three independent replicates for data shown in Figure 2B and four independent replicates for data shown in Supplementary Figure S4), and data were represented as mean RQ (relative quantification) ± s.d. Statistical analyses were calculated using Student’s t-test.

### Microscopy

#### Imaging of the lipid droplets and the lysosomal-related organelle in the intestine

The *C. elegans* strain LIU1 harboring the lipid droplet (LD) marker DHS-3::GFP was synchronized using hypochlorite disruption as described, and the synchronized gravid young adults (50–52 h for DA, 52–53 h for HG-DA, 56–57 h for OP, and 59–61 h for HG-OP post-L1 feeding) were examined. One day prior to the imaging experiment, worms were shifted to 5.5-cm NGM plates seeded with the same diet mixed with 200 μL of 10 μM LysoTracker Red and incubated in the dark. Animals were harvested from the plates in M9 and anesthetized in 1 mM levamisole, and samples were placed on a 2% agarose pad (15 × 15 mm) for imaging. We included N2 as a negative control for all of our imaging experiments. Images were acquired on a Leica Stellaris 8 inverted microscope system equipped with a Plan Apo 63x/1.2 water immersion objective lens, GFP 1 (Ex 489, Em 493–532), and LysoTracker Red 2 (Ex561, Em 564–651). A single plane of 3 μm was captured. Three independent replicates were conducted (n > 10/ea), and representative data are shown.

#### Imaging of LDs in the oocyte

To image LDs in the gonad, worms were synchronized by picking ∼100 L4 to a new plate. Young adult animals were harvested from plates, washed with M9 buffer, then stained in the dark with 13.3 μg/mL BODIPY 493/503 in 0.1% DMSO for 45 min, followed by washing with M9 buffer thrice. After being anesthetized in 20 mM tetramisole, samples were placed on a 2% agarose pad (15 × 15 mm) for imaging using a Leica Stellaris 8 inverted microscope system equipped with a Plan Apo 40x/1.1 water immersion objective lens, and GFP (Ex 481, Em 495–596). We captured every 0.3-μm z-step for a total thickness of 5 μm, followed by maximal projection. Three independent replicates were conducted. To quantify LDs in the −4 oocytes, the maximal displayed value for the image was set to 9,000 using ImageJ. We outlined the entire cell and the nucleus to calculate their mean intensities separately. The mean intensity of the LDs was then determined by subtracting the mean intensity of the nucleus from that of the cell.

#### Imaging of yolk proteins in the oocyte and the intestine

To image the *C. elegans* strain RT130 harboring VIT-2::GFP, worms were synchronized by picking ∼100 L4 to a new plate. Young adult animals were harvested and washed with M9 buffer, and after being anesthetized in 20 mM tetramisole, samples were placed on a 2% agarose pad (15 mm × 15 mm) for imaging using a Leica Stellaris 8 inverted microscope system equipped with a Plan Apo 40x/1.1 water immersion objective lens and GFP (Ex 487, Em 494–598). We captured every 2.28-μm z-step for a total of six planes, followed by maximal projection. Three independent replicates were conducted. To quantify VIT-2::GFP intensity in the −1 oocytes, we use a single Z image, and the maximal displayed value for the image was set to 100 using ImageJ. We outlined the entire cell and the nucleus to calculate their mean intensities separately. The mean intensity of the VIT-2::GFP was then determined by subtracting the mean intensity of the nucleus from that of the cell.

#### Imaging of H2B::GFP

To image the *C. elegans* strain MAH74 harboring H2B::GFP, worms were synchronized by picking ∼100 L4 to a new plate. Young adult animals were harvested from plates and washed with M9 buffer, and after being anesthetized in 20 mM tetramisole, samples were placed on a 2% agarose pad (15 × 15 mm) for imaging using a Leica Stellaris 8 inverted microscope system equipped with a Plan Apo 40x/1.1 water immersion objective lens and GFP (Ex 488, Em494–598). A total of 31 planes were captured with a 0.6 μm z-step size, followed by maximal projection. Three independent replicates were conducted, and representative data are shown.

### Imaging of mNG::LET-60, mNG::MPK-1, and Pmex-5::BFP

All quantified data were imaged in at least three independent replicates. All germline images were performed on a spinning disk confocal system that uses a Nikon 60 × 1.2 NA water objective, a Hamamatsu C15440 ORCA-Fusion BT Digital Camera, and a Yokogawa CSU-X1 Confocal Scanner Unit. Nikon’s NIS imaging software was applied to capture the images. The image data were processed using ImageJ/FIJI with the Bio-formats plugin (National Institutes of Health) (Linkert et al., 2010; Schindelin et al., 2012). The imaged worms were day-1 adult hermaphrodites (24 h post-mid-L4). The fluorescent signal intensity was quantified within a defined area (40 pixels × 40 pixels), and three independent regions (40 pixels × 40 pixels) were also measured in each image as background controls. The final quantification ratio was standardized by dividing the fluorescent intensity of the selected area by the average background intensity.

#### Lipid extraction and lipidomic analysis

*C. elegans* were synchronized using hypochlorite disruption as described. The synchronized young adults (50 h for DA, 52 h for HG-DA, 55 h for mix 9:1, 56 h for OP, 56 h for mix 3:1, and 62 h for HG-OP post-L1 feeding) were harvested for lipid extraction and analysis. After being washed with sterilized water and pelleted, worms were immediately frozen in liquid nitrogen (∼25,000 young adults per diet treatment). Samples were thawed and lysed using the TissueLyser II (QIAGEN, Hilden, Germany) at a frequency of 30 Hz for 30 s, then submerged in liquid nitrogen to re-freeze, and thawed for 2 min in a water bath; this process was repeated for eight cycles. A small amount of lysate was quantified using the Bradford assay to measure total protein concentration, which was used for normalization. Lipid extraction was performed following the Folch method (Folch et al., 1957). Approximately 150 μL of worm lysates were mixed in a conical glass tube with 2 mL chloroform/methanol (2:1 v/v) and an internal control (FA 13:0) and incubated overnight at 4 °C. Hajra’s solution (0.2 M H_3_PO_4_ and 1 M KCl) was added to the lysate. The mixture was spun down for 10 min at 10,000 g to separate aqueous and organic layers. The organic layer (lower phase) was isolated and dried using a Thermo SpeedVac concentrator SPD111V (Thermo Scientific). The aqueous layer was treated with chloroform to re-separate the organic phase and dried again using a Thermo SpeedVac concentrator SPD111V (Thermo Scientific). Dried samples were dissolved in 150 μL chloroform/methanol (2:1 v/v).

To perform lipidomic analysis using liquid chromatography/mass spectrometry/mass spectrometry (LC/MS/MS), a linear Orbitrap Elite Ion Trap-Orbitrap mass spectrometer (Thermo Fisher Scientific) coupled online with an ACQUITY UPLC/UHPLC System (Waters Corporation, Milford, MA, United States) was used. Lipid samples (4 µL) in chloroform/methanol (2:1 v/v) were separated using an ACQUITY UPLC CSH C18 column (1.8 µm, 2.1 mm × 100 mm, Waters) at a flow rate of 0.5 mL/min, with a gradient from 40% to 99.9% solvent B over 0–10 min, holding at 99.9% B for 2 min, followed by re-equilibration at 40% B. Solvent A consists of acetonitrile/water (40:60) with 10 mM ammonium acetate (pH 5.0), and solvent B consists of isopropanol/acetonitrile (90:10) with 10 mM ammonium acetate (pH 5.0). The MS was operated with either positive or negative ion modes using the full Fourier transform–mass spectrometry scan at 100–1,200 m/z, with a resolution of 60,000. Target search and the quantification were performed using Xcalibur software (Thermo Scientific), and the final statistical analysis was performed using Excel (Microsoft).

FAs in animals were profiled using gas chromatography/mass spectrometry (GC/MS). Lipid samples prepared in chloroform/methanol (2:1 v/v) as described were derivatized into fatty acid methyl esters (FAMEs). In brief, 50 μL lipid samples were mixed with 14% BF3 in methanol and heated up at 95 °C for 10 min. After cooling to room temperature, the samples were mixed with benzene and heated to 95 °C for 30 min. Once cooled again to room temperature, the samples were treated with sterile water and petroleum ether and centrifuged at 10,000 g for 3 min to separate the organic and aqueous phases. The organic phase was isolated and dried using a Thermo SpeedVac concentrator SPD111V (Thermo Scientific). Dried samples were dissolved in 100 μL chloroform/methanol (2:1 v/v) and loaded for GC/MS using an Agilent 7890A GC System equipped with a 5977B inert MSD. An aliquot of 1 μL of derivatized samples was injected using the inlet mode splitless, and FAMEs were separated using an Agilent DB-5MS Column (30 m × 0.25 mm ×0.25 μm) at a flow rate of 1.1 mL/min. The inlet temperature was 250 °C. The column temperature was initially held at 80 °C for 1 min, followed by an increase to 128 °C at a rate of 8 °C/min, to 188 °C at 10 °C/min, to 222 °C at 2 °C/min, to 228 °C at 3 °C/min, to 278 °C at 5 °C/min, to 305 °C at 4.5 °C/min, and finally to 310 °C at 5 °C/min, where it was held for 6 min. The data were acquired in scan mode (27–540 m/z) at 70 eV. Quantification and target search were performed using Agilent ChemStation data analysis software, and the NIST Mass Spectral Search Library was used. Statistical analysis was performed in Excel (Microsoft). The data presented in this study are summarized from at least four independent replicates, each with 2–-3 technical replicates.

#### Metabolomic analysis

We used the two-phase extraction method to isolate aqueous-phase metabolites for metabolomic analysis. *C. elegans* were synchronized using hypochlorite disruption as described. The synchronized young adults (50 h for DA, 52 h for HG-DA, 55 h for mix 9:1, 56 h for OP, 56 h for mix 3:1, and 62 h for HG-OP post-L1 feeding) were harvested for metabolomic analysis. After being washed with sterilized water and pelleted, worms were immediately frozen in liquid nitrogen (∼25,000 young adults per diet treatment). Worm lysates and extraction were carried out as described in the lipid extraction method, except that the aqueous phase (upper phase) was recovered. Samples were dried and resuspended in 70 μL of 50% methanol.

For running at negative ion mode, solvent A2 consists of 50% acetonitrile/20 mM ammonium acetate, pH 9.0, and solvent B2 consists of 90% acetonitrile/20 mM ammonium acetate, pH 9.0. The column temperature is set at 30 °C. The running program started from 99.9% B to 65% B over 1 min, 65% B to 55% B over 1–5.5 min, 55% B to 0.1% B over 5.5–7.5 min, with 0.1% B over 7.5–9.5 min, 0.1% B to 99.9% B over 9.5–10 min, and 99% B over 10–12 min. For running at positive ion mode, solvent A2 consists of 50% acetonitrile/20 mM ammonium formate, pH 3.0, and solvent B2 consists of 90% acetonitrile/20 mM ammonium formate, pH 3.0. The program started from 99.9% B to 90% B over 2 min, 90% B to 50% B over 2–6 min, 50% B to 0.1% B over 6–7.5 min, with 0.1% B over 7.5–9.5 min, 0.1% B to 99.9% B over 9.5–10 min, and 99% B over 10–12 min. The MS was operated with either positive or negative ion modes using the full Fourier transform–mass spectrometry scan at 70–1,000 m/z, with a resolution of 60,000.

The LC-MS data were processed using MS-DIAL software (version 4.90) (Tsugawa et al., 2020) against a combined spectral library composed of the MS-DIAL MSP spectral kit and the NIST 20 Tandem Mass Spectral Libraries. For peak detection, the minimum peak height and peak width were set at 30,000 amplitude and 10 scans, respectively. Peak smoothing was achieved using a linear weighted moving average over four scans. For metabolite identification, mass tolerances were set at 0.008 Da for MS and 0.05 Da for MS/MS, with a score cutoff of 75. The peak areas were subsequently exported for quantification using Excel (Microsoft). Further targeted search and quantification for metabolites include choline ([M + H+] = 104.107, RT1.63), betaine ([M + H+] = 118.0868, RT3.21), phosphocholine ([M + H+] = 186.0895, RT5.67), cytidine 5′-diphosphocholine ([M + H+] = 489.1152, RT6.91), glycerophosphocholine ([M + H+] = 258.1106, RT5.4), S-adenosyl-L-methionine ([M + H+] = 399.1451, RT6.81), S-(5′-adenosyl)-L-homocysteine ([M + H+] = 385.1294, RT5), L-methionine ([M + H+] = 150.0589, RT3.56), and cystathionine ([M + H+] = 223.0753, RT6.77). The data presented in this study are summarized from at least four independent replicates, each with 2–3 technical repeats.

#### Determination of the progeny number and hatch ratio

**FIGURE 1:**
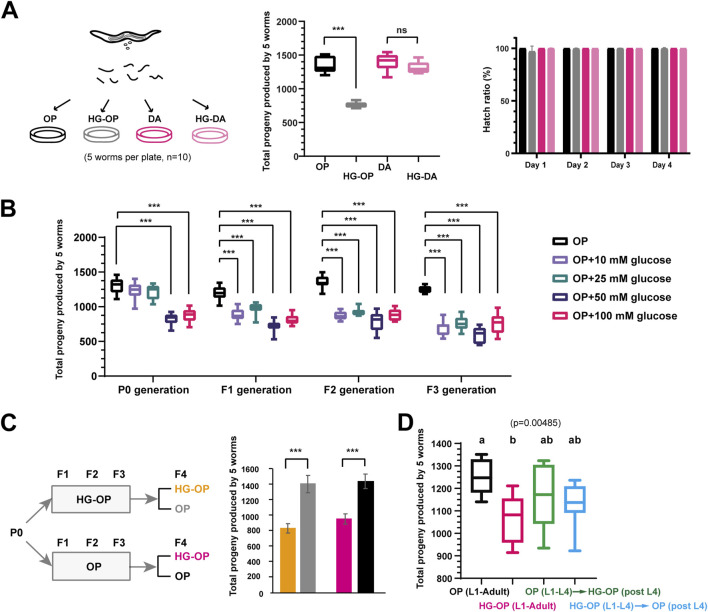
*C. elegans* fed *Escherichia coli* OP50 but not *C. aquatica* DA1877 exhibit reduced fecundity under high-glucose conditions. **(A)** Synchronized L1 larvae of the *C. elegans* N2 strain were fed on NGM plates or NGM plates supplemented with 100 mM glucose (HG), which were seeded with *E. coli* OP50 (OP) or *C. aquatica* DA 1877 (DA). L4-stage worms were transferred to new plates with every five worms per plate to determine their total progeny number. The reproductive output is shown as the sum of total progeny numbers by five worms and is plotted using Box and Whisker diagrams. The hatch ratio was shown from day 1 to day 4 adult after the L4 stage; Statistical analysis was performed using two-tailed Student’s *t*-test (***, *p* < 0.001; ns, not significant); n = 10. **(B)** Synchronized L1 larvae of the *C. elegans* N2 strain were fed on NGM plates supplemented with different concentration of glucose. The P0 progenies were synchronized to prepare L1-stage worms for F1 generation analysis. The sum of total offspring numbers from five worms was plotted using Box and Whisker diagrams. Statistical analysis was performed using two-tailed Student’s *t*-test (***, *p* < 0.001). n = 10. **(C)** *C. elegans* were fed on NGM plates seeded with *E. coli* OP50 either in the absence (OP) or presence of 100 mM glucose (HG-OP) from P0 to F3 generations. The P0 progenies were synchronized to prepare L1-stage worms for F1 generation analysis. The sum of total offspring numbers from five worms in the F4 generation was plotted as mean+/−s.d. Statistical analysis was performed using two-tailed Student’s *t*-test (***, *p* < 0.001; ns, not significant). n = 10. **(D)** *C. elegans* N2 strains were fed NGM plates either in the absence (OP) or in the presence of 100 mM glucose (HG-OP) to determine their total progeny number (L1–adult). The results were compared with *C. elegans* fed on OP plates until the L4 stage and then transferred to new HG-OP plates, along with worms fed on HG-OP plates until the L4 stage and then transferred to new OP plates. The total offspring numbers from five worms was plotted using Box and Whisker diagrams. Statistical analysis was performed using one-way ANOVA; n = 10.

*C. elegans* eggs were isolated from gravid adults using hypochlorite disruption (0.5 N NaOH and 1% NaOCl), followed by several washes with M9 buffer. After overnight incubation in M9 buffer, synchronized L1 larvae were transferred to OP, HG-OP, DA, and HG-DA plates. At the mid-L4 stage, five hermaphrodites were picked and transferred to new 5.5-cm plates containing the same diet, with a total of 10 replicates. The animals were transferred to fresh plates daily for the next four consecutive days. The number of hatched and unhatched progeny was counted 24 h after removing the adults from the plate. Progeny produced over five consecutive days were summed to represent the total reproductive output. Hatch ratio= (the number of hatched animals/the total number of hatched and unhatched animals) *100%. To assay the reproductive output across generations, we prepared synchronized L1 larvae from adults of the previous generation grown on designated plates as described and transferred them to the appropriate plates according to the experimental scheme shown in Figures 1B,C. At the mid-L4 stage, five hermaphrodites were picked and transferred onto new 5.5-cm plates with the same diet, with a total of 10 replicates. Progeny counts and reproductive output were assessed as described.

To compare reproductive output between mated and unmated animals, synchronized *C. elegan*s L1 larvae were prepared as described above. At the mid-L4 stage, 50 hermaphrodites were picked and transferred to new 5.5-cm plates containing the same diet, and another 50 hermaphrodites were placed on plates with 50 male worms. After 24 h, five hermaphrodites in the two groups were transferred to new 5.5-cm plates containing the same diet, with a total of 10 replicates. The animals were transferred to fresh plates daily for the next four consecutive days. Progeny counts and reproductive output were assessed as previously described.

#### Dietary supplementation of FAs, choline, betaine, methionine, and B12

To address the effect of FA supplementation in the HG-OP diet, palmitic acid (FA16:0, Sigma # P0500), oleic acid (FA18:1n9, Sigma # O1383), cis-vaccenic acid (FA18:1n7, Sigma # V0384), and α-linolenic acid (FA18:3n3, Sigma # L2376), dissolved in ethanol, were added to NGM containing 100 mM glucose and 0.2% Brij58 to achieve a final FA concentration of 300 μM. *C. elegans* eggs were isolated from gravid adults using hypochlorite disruption (0.5 N NaOH and 1% NaOCl), followed by washing with M9 buffer. After overnight incubation in M9 buffer, synchronized L1 larvae were transferred to various FAs or 100 mM glucose NGM plates containing 0.2% Brij58, seeded with OP. To address the effects of choline, betaine, methionine, and vitamin B-12 supplementation in the HG-OP diet, 50 μM choline (Sigma #C7527), 50 μM betaine (Sigma #107437), 5 μM methionine (Sigma #M9625), and 6.4 nM vitamin B12 (cobalamin, Sigma #C0884) were added to NGM containing 100 mM glucose to prepare the plates. Five mid-L4 hermaphrodites were picked onto new 5.5-cm plates containing the same diet state for 10 repeats, and the animals were transferred to new plates on each of the following four consecutive days. Progeny counts and reproductive output were assessed as previously described. Data analysis, including one-way ANOVA and graph generation, was performed using GraphPad Prism. Each of Figures 4E, 6F,G represents data from two independent trials that yielded consistent results; however, only one representative dataset is shown.

### Statistical analysis

For most experiments, statistical analysis was performed using a two-tailed Student’s *t*-test (*, *p* < 0.05; **, *p* < 0.01; ***, *p* < 0.001), and data are presented as the mean ± SD. One-way ANOVA was also used for comparisons involving more than three independent groups.

## Results

### Bacteria diets differentially affect *C. elegans* fecundity under high-glucose conditions

Several recent reports have indicated that *C. elegans* fed high-glucose diets exhibit a reduced brood size (Alcantar-Fernandez et al., 2018; Choi, 2011; Lee et al., 2009). Intriguingly, among the various bacterial diets tested, we found that *C. elegans* fed the *C. aquatica* DA1877 diet supplemented with 100 mM glucose from the L1 stage maintained a total progeny number comparable to those fed the same diet without glucose. In contrast, *C. elegans* fed the standard *E. coli* OP50 diet supplemented with 100 mM glucose exhibited a reduced progeny number compared to those fed the same diet without glucose, although the hatch ratio remained close to 100% (Figure 1A). The decrease in progeny number in worms fed OP50 is attributed to glucose, as evidenced by the glucose concentration-dependent reduction and the cumulative effect observed in worms exposed to low glucose concentrations over multiple generations (Figure 1B).

When *C. elegans* were fed the OP50 diet supplemented with 100 mM glucose for three generations and then shifted as L1 progeny to OP50 with or without 100 mM glucose, a recovery to healthy reproductive output was observed on the *E. coli* OP50 diet without glucose (Figure 1C), indicating that the glucose-induced decrease in fecundity is not heritable. Moreover, the deleterious effects of high glucose persisted throughout the worms’ life history on the OP50 diet: animals exposed to 100 mM glucose either from L1 to L4 or after L4 exhibited intermediate reproductive output, falling between those fed high glucose throughout life and those never exposed to high glucose (Figure 1D). In summary, the *E. coli* OP50 diet represents a “harmful” high-glucose artificial diet that reduces fecundity in *C. elegan*s, while the *C. aquatica* DA1877 diet represents a “protective” high-glucose artificial diet that mitigates the harmful effects of high glucose on *C. elegans*.

### High-glucose diets differentially affect the expression of genes involved in lipid metabolism

**FIGURE 2:**
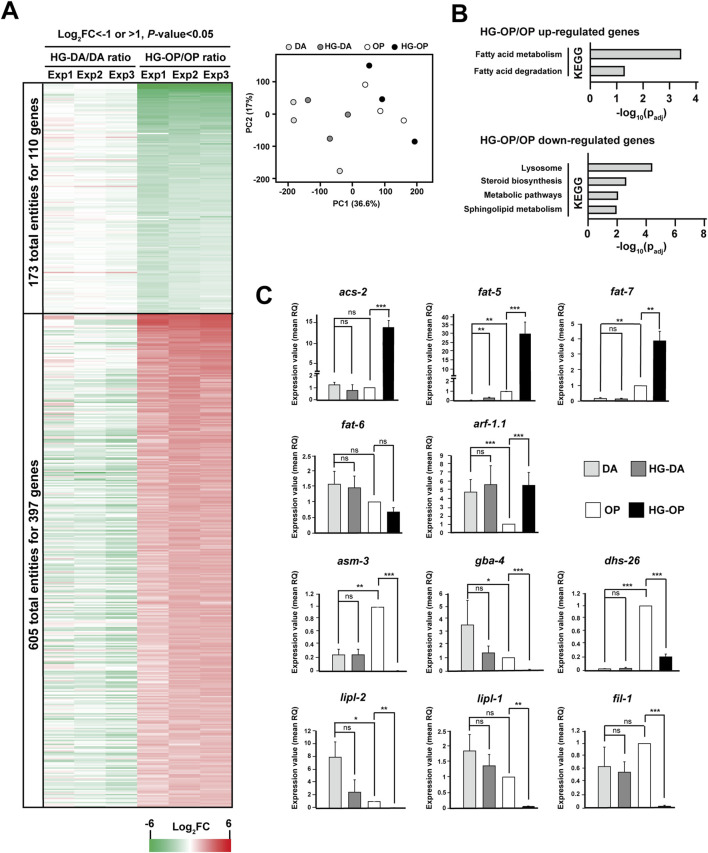
High-glucose diets lead to varying gene expression patterns in *C. elegans* **(A)** The *C. elegans* N2 strains fed different diets were harvested and subjected for microarray analysis. The displayed genes were those with Log_2_FC < −1 (green) or >1 (red), and *P*-value < 0.05 identified from *C. elegans* fed HG-OP compared with OP. Data represent three independent experiments. The PCA plot summarizing the results of the three independent experiments is displayed on the right. **(B)** The 397 genes that were upregulated and the 110 genes that were downregulated in worms fed HG-OP compared with those fed OP were subjected to KEGG pathway analysis. **(C)** *C. elegans* N2 strains fed different diets were harvested, and the isolated mRNAs were subjected for qPCR analysis. Genes as indicated were analyzed, and the fold change is shown. The gene expression level of OP was set as 1. Statistical analysis was performed using two-tailed Student’s *t*-test (*, *p*< 0.05; **, *p*< 0.01; ***, *p*< 0.001; ns, not significant), and the data are presented as the mean RQ ± s.d.; n = 3.

To explore the intricate interactions between diets and high glucose, we first applied transcriptomic analysis using microarrays to gain insights. By comparing fold changes in gene expression, we identified a list of genes whose expression levels were either upregulated or downregulated specifically in worms fed the high-glucose *E. coli* OP50 diet (termed HG-OP hereafter) compared with those fed the *E. coli* OP50 diet alone (termed OP hereafter) (Figure 2A). In contrast, only a subtle difference was observed when comparing worms fed the high-glucose *C. aquatica* DA1877 diet (termed HG-DA hereafter) with those fed the *C. aquatica* DA1877 alone (termed DA hereafter). Notably, a number of genes significantly affected by the addition of high glucose to *E. coli* OP50 are involved in lipid metabolic processes and lysosomes (Ladage et al., 2016), as depicted by the KEGG pathway analysis (Figure 2B).

We conducted qPCR to assess the expression of genes involved in lipid metabolism. *acs-2* and *fat-5* were among the most upregulated genes under HG-OP compared with OP alone, while minimal changes were observed under HG-DA compared with DA (Figure 2C). The *acs-2* gene encodes fatty acyl-CoA synthetase, and the *fat-5* gene encodes one of *C. elegans* Δ-9 FA desaturases, producing FA16:1n7 that can be elongated to FA18:1n7. *fat-7*, *the* Δ−9 FA desaturase gene catalyzing FA18:1n9 production, was largely induced under HG-OP compared with OP, whereas the other Δ−9 FA desaturase gene, *fat-6*, which also catalyzes FA18:n9 production, was not induced (Figure 2C). Most other genes in the FA biosynthesis pathway, however, remained largely unaffected by HG-OP as in HG-DA (Supplementary Figure S1). The most downregulated gene specific to HG-OP compared with OP is *asm-3*, while no change was observed with HG-DA compared with DA (Figure 2C). *asm-3* encodes acid sphingomyelinase, contributing phosphocholine for PC synthesis. Although most genes in the sphingolipid biosynthesis pathway were not significantly regulated by HG-OP compared with OP (Supplementary Figure S1), *gba-4* showed a similar pattern of regulation to *asm-3*. Additionally, the lysosomal lipase genes *lipl-1* and *lipl-2*, involved in fat catabolism, were downregulated, as were *fil-1* and *dhs-26* (Figure 2C). Overall, these data identified a trend of elevated FA metabolism and reduced lysosomal activities specific to HG-OP diets, highlighting lipid metabolic processes as one major hub for the differential effects of high-glucose diets (Garcia et al., 2015; Ladage et al., 2016).

### *C. elegans* fed the high-glucose OP50 diet show increased LD size and elevated vitellogenin levels

**FIGURE 3:**
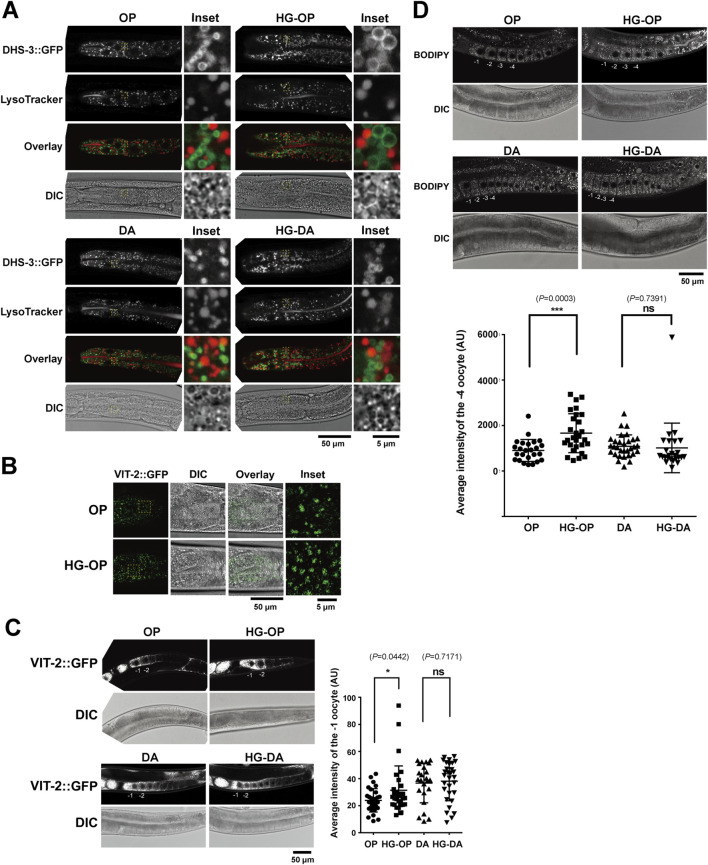
*C. elegans* fed HG-OP exhibit increased LD size and vitellogenin levels in the intestine and oocytes. **(A)** The *C. elegans* strain LIU1, harboring the LD marker DHS3::GFP, was fed different diets, stained with LysoTracker Red, and imaged using confocal microscopy. The insets show the enlarged regions of interest (ROIs). **(B)** The *C. elegans* strain RT130, harboring VIT-2::GFP, was fed different diets and imaged using confocal microscopy, focusing on the intestinal cells. The insets show the enlarged ROIs. **(C)** Same as B, except with a focus on oocytes. The averaged signal intensity of VIT-2::GFP of the −1 oocyte was compared. Statistical analysis was performed using two-tailed Student’s *t*-test (*, *p*< 0.05; ns, not significant). **(D)** The *C. elegans* N2 strains fed on different diets were stained with BODIPY 493/503 and imaged using confocal microscopy. The averaged signal intensity of BODIPY 493/503 in the −4 oocyte was compared. Statistical analysis was performed using two-tailed Student’s *t*-test (***, *p*< 0.001; ns, not significant).

Glucose metabolism involves an intricate network of pathways (Watts and Ristow, 2017). In complex organisms, excess glucose is converted to TAG encapsulated within the storage organelles known as the LD. Additionally, the catabolic organelles—lysosomes—play pivotal roles in the maintenance of cellular lipid homeostasis. The *C. elegans* intestine performs functions analogous to mammalian digestive traits, liver, and fat-storage tissues (Dimov and Maduro, 2019). LDs and the LROs are abundant metabolic organelles in the *C. elegans* intestine (Hermann et al., 2005; Mak, 2012; Soukas et al., 2013). To assess their health, we examined a strain expressing the LD marker DHS-3::GFP, a protein that localizes exclusively to intestinal LDs (Zhang et al., 2012), and stained with LysoTracker Red, which labels the LROs, in animals fed different diets with and without high glucose. In line with a recent finding, we observed that *C. elegans* fed DA accumulated more LROs than those fed OP, and OP-fed worms had slightly larger LDs than those fed DA, reflecting a dietary effect (Figure 3A) (Han et al., 2024). We also found that *C. elegans* fed HG-OP displayed an increase in size and the number of LDs labeled by DHS-3::GFP, but with no noticeable change for the LROs (Figure 3A). In contrast, *C. elegans* fed HG-DA showed slightly larger LDs than those fed on a DA diet alone, yet their size remained noticeably smaller than the size of those fed HG-OP and was similar to the size of those observed in worms fed OP alone. These results indicate that *C. elegans* fed the HG-OP diet accumulate excess fat in intestinal LDs.

The *C. elegan*s intestine synthesizes yolks, which structurally and functionally mimic mammalian lipoproteins, facilitating the transfer of lipids/FAs from the intestine to oocytes during reproduction (Kimble and Sharrock, 1983; Perez and Lehner, 2019). Using VIT-2::GFP as a yolk marker, microscopy results identified that *C. elegans* fed the HG-OP diet showed increased VIT-2::GFP puncta in the intestine (Figure 3B). In *C. elegans*, VIT-2::GFP is most abundantly found in the oocytes. Quantification indicated stronger VIT-2::GFP signals in the −1 oocyte of worms fed the HG-OP diet than in those fed OP, although no change in VIT-2:GFP signal intensity was detected in worms fed HG-DA compared with those fed DA (Figure 3C). Given that VIT-2 is a yolk protein involved in reproduction and is typically produced in response to certain metabolic conditions (Goszczynski et al., 2016), its higher levels could be linked to reproductive changes or alterations in fat storage and transport.

In the oocyte, LDs are also prominent organelles that work together with yolks to regulate lipid metabolism necessary for germ cell and embryonic development in *C. elegans* (Chen et al., 2016; Vrablik et al., 2015). We monitored oocyte LDs using BODIPY 493/503 staining (Bai et al., 2020) and found significantly higher BODIPY 493/503 signals in worms fed the HG-OP diet than in those fed OP, in contrast to no change in worms fed HG-DA compared with those fed DA (Figure 3D). Together, we conclude that animals fed the HG-OP diet accumulated excess TAG and yolk in both intestinal cells and developing oocytes.

### *C. elegans* fed the *Escherichia coli* OP50 diet under high-glucose conditions exhibit altered FA homeostasis

**FIGURE 4:**
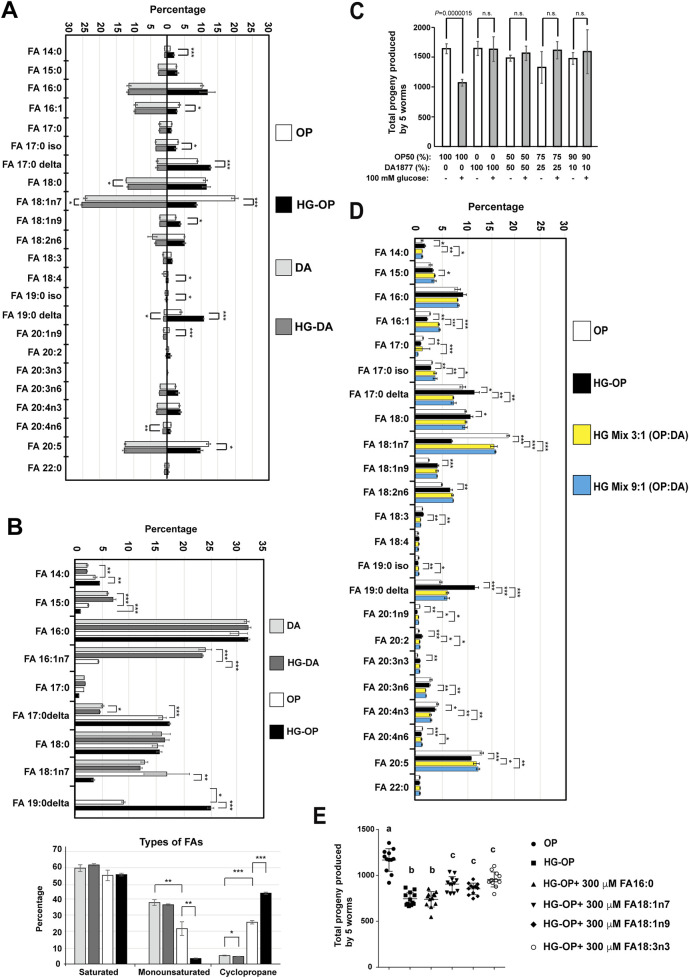
*C. elegans* fed HG-OP but not HG-DA diets show altered FA homeostasis. **(A)** Lipids extracted from the *C. elegans* N2 strain fed different diets were converted to FA methyl esters and analyzed using gas chromatography–mass spectrometry (GC-MS). The percentage of each FA species was compared, and the data are presented as the mean ± s.d. Statistical analysis was performed using two-tailed Student’s *t*-test (*, *p*< 0.05; **, *p*< 0.01; ***, *p*< 0.001). n = 4. **(B)** Same as A, except that FAs were analyzed from bacteria. The FA species shown in the upper panel were grouped and displayed according to their classification as saturated, monounsaturated, or cyclopropane FAs, as summarized in the lower panel. Statistical analysis was performed using two-tailed Student’s *t*-test (*, *p*< 0.05; **, *p*< 0.01; ***, *p*< 0.001). n = 4. **(C)** Reproductive output was determined for the *C. elegans* N2 strain fed on different mix diets as indicated, and data were presented as the mean+/-s.d. Statistical analysis was performed using two-tailed Student’s *t*-test (ns, not significant); n = 10. **(D)**Same as **(A)**HG mix 3:1 (OP:DA) and HG mix 9:1 (OP:DA) refer to mixtures of three or nine volumes of OP with one volume of DA, respectively, seeded on NGM plates containing 100 mM glucose. **(E)**Synchronized L1 larvae of the *C. elegans* N2 strain were fed *E. coli* OP50 on NGM plates containing 0.2% Brij58 in the absence (OP) or presence of 100 mM glucose (HG-OP), supplemented with 300 μM FAs as indicated. Reproductive output was determined, and data are presented as the mean+/−s.d. Statistical analysis was carried out using one-way ANOVA.

To investigate how lipid metabolic processes mediate the effects of a high-glucose diet, we extracted lipids from young adult animals, derivatized them to prepare FA methyl esters, and subjected the samples to FA profiling using gas chromatography/mass spectrometry (GC/MS). Although OP and DA were different bacterial species, *C. elegan*s fed DA and OP displayed a very similar FA profile, with major differences in only three FAs, namely, FA17:0Δ, FA19:0Δ, and FA16:1n7 (Figure 4A). Worms fed either of the two bacterial diets showed little to no change in healthy fecundity, implying that these FAs, at least at the current levels, might not directly contribute to fecundity reduction.

When comparing worms fed HG-OP with those fed OP, we identified a significant induction in the level of cyclopropane FAs (FA17:0Δ and FA19:0Δ) and a reduction in the monounsaturated FAs (MUFAs), FA18:1n7 and FA16:1n7; in contrast, little to no changes were observed in FAs in worms fed HG-DA compared to those fed DA alone (Figure 4A). Intriguingly, these are lipids that worms may acquire from their bacterial diets. This raises the possibility that the high-glucose dietary effect may be attributed to bacteria processing high-glucose, which prompted us to harvest dietary bacteria from the NGM plates and analyze their FA profiles. Although saturated FA (SFA) levels are similar in both bacteria, DA has more MUFAs, especially FA16:1n7, while OP has more cyclopropane FAs, FA17:0Δ and FA19:0Δ (Figure 4B). Importantly, HG-OP, but not HG-DA, significantly reduced MUFAs (FA18:1n7 and FA16:1n7) and increased FA19:0Δ, indicating that high glucose readily impacts bacterial FA homeostasis in OP but not in DA.

To investigate whether DA could counteract the fecundity reduction observed in *C. elegans* on an HG-OP50 diet, we mixed small portions of DA (1/2, 1/4, and 1/10) into the HG-OP50 diet. Since DA preserves fecundity under high-glucose conditions, we hypothesize that it may restore fecundity in animals fed HG-OP by normalizing lipid homeostasis. Worms fed all three mixed diets restored normal progeny number (Figure 4C). FA profiling data further indicate that animals fed the high-glucose mix diets, HG mix 3:1 (DA:OP) and HG mix 9:1 (DA:OP), display the MUFA FA18:1n7 and the cyclopropane FA19:0Δ comparable to those in worms fed OP (Figure 4D). Surprisingly, there was minimal difference in the overall FA profiles between the two mixed diet conditions, even though the ratio of DA mixed into HG-OP varied. Additionally, we observed that the trends in MUFA and cyclopropane FA levels in the two high-glucose mixed bacterial diets mirrored those observed in *C. elegans* (Supplementary Figure S2).

To clarify the exact contributions of FAs to the fecundity of *C. elegans*, we investigated whether supplementing HG-OP with various types of FAs could restore the reduced fecundity in *C. elegans*. The results show that adding MUFA (18:1n7 and 18:1n9) or PUFA (18:3n3), but not SFA16:0, slightly elevated the progeny number in worms fed HG-OP (Figure 4E). Given that FA 18:1n7 is the most reduced FA species in the HG-OP diet and it did not have a specific effect compared with other MUFA or PUFA supplementations, it seems likely that the fecundity decrease in *C. elegans* is not merely due to altered FAs consumed from dietary bacteria. In other words, the FA variation is associated with decreased fecundity, most likely reflecting dietary effects rather than being the primary cause.

### *C. elegans* fed the *Escherichia coli* OP50 diet under high-glucose conditions show reduced PC abundance

**FIGURE 5:**
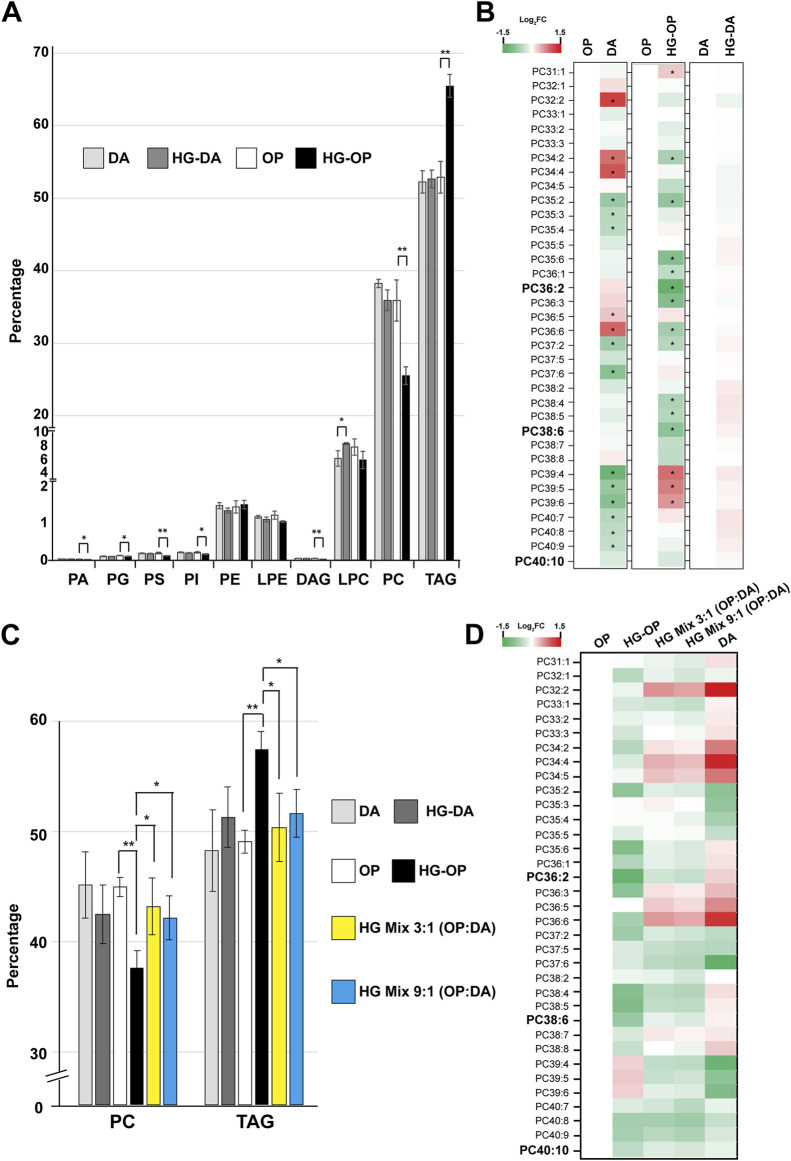
*C. elegans* fed HG-OP but not OP, HG-DA, or DA diets exhibit reduced PC and elevated TAG. **(A)** Lipids were extracted from the *C. elegans* N2 strain fed different diets and analyzed using liquid chromatography–mass spectrometry (LC-MS). The total abundance of various types of glycerolipids was summed, and the percentage distribution was shown. Statistical analysis was performed using two-tailed Student’s *t*-test (*, *p* < 0.05; **, *p* < 0.01; ***, *p* < 0.001); n = 4. PA, phosphatidic acid; PG, phosphatidylglycerol; PS, phosphatidylserine; PI, phosphatidylinositol; PE, phosphatidylethanolamine; LPE, lysophosphatidylethanolamine; DAG, diacylglycerol; LPC, lysophosphatidylcholine; PC, phosphatidylcholine; TAG, triacylglycerol. **(B)** The abundance of each PC species in worms fed different diets were quantified, and the relative Log_2_ FC (fold change), comparing samples to OP or DA as shown, was displayed in heat maps. PC38:6, PC36:2, and PC40:10 are the top three most abundant PC species in worms fed OP. **(C)** Same as A, except that only the abundance of PC and TAG in total glycerolipids was compared. **(D)** The abundance of each PC species in worms fed different diets were quantified, and the relative Log_2_ FC (fold change), comparing samples to OP as indicated, was shown in heat maps.

Given that FAs serve as fundamental constituents of various cellular lipids (Watts and Ristow, 2017), we next conducted lipidomic analysis using liquid chromatography/mass spectrometry (LC/MS) to delve deeper into the various lipid species (Bai et al., 2020). We identified over 500 species of glycerolipids, including glycerophospholipids, diacylglycerols (DAGs), and TAGs, and observed a number of changes in the lipidome specific to worms fed HG-OP50 (Supplementary Figure S3). Strikingly, worms fed HG-OP display an overall decrease in the major membrane lipid PC and an increase in the major storage lipid TAG (Figure 5A). PC and TAG represent two primary *C. elegans* glycerolipids, constituting approximately 36% and 52% of the total glycerolipids, respectively (Figure 3B). Although worms fed either OP or DA diets exhibit similar overall PC levels (Figure 5A), the composition of their PC species differs (Figure 5B), implying distinct contributions from specific metabolic pathways. Interestingly, we identified that PC reduction in *C. elegans* fed HG-OP is associated with specific PC species, such as the most abundant PC species PC38:6 (Supplementary Figure S5). This lipid species is maintained at nearly identical amounts in worms fed either OP or DA diets. However, this lipid species was reduced to approximately 40% of the normal level in animals fed HG-OP, supporting overall PC reduction (Figure 5B). PC36:2 showed a similar reduction pattern to that of PC38:6. Additionally, we found that many PC species that changed in HG-OP worms exhibited an inverse correlation with those in DA-fed worms compared to OP (Figure 5B). These data imply that metabolic decisions differ between DA and OP diets, likely being modulated inversely under HG-OP conditions, contributing to changes in PC and TAG levels. It also suggests that this metabolic decision may be regulated by factors present in the DA diet under high-glucose conditions, preventing lipid perturbation in HG-DA by maintaining PC abundance.

To better investigate the effects of PC, we further compared PC abundance in the two high-glucose mixed diets, HG Mix 3:1 (OP:DA) and HG Mix 9:1 (OP:DA), with that in OP, HG-OP, DA, and HG-DA. The results show that both mixed-diet conditions were adequate to restore the overall PC and TAG levels similar to OP, DA, and HG-DA (Figure 5C). In addition, both mixed diets restore the overall PC profile toward a pattern resembling that of DA compared with that of OP (Figure 5D). Intriguingly, as for FA profiling data, there was not much difference in the overall lipidome between the two mixed-diet conditions, further suggesting that the presence of a dilutable factor in the DA is sufficient to adjust the normal lipid homeostasis of HG-OP conditions.

Taking advantage of the mixed-diet experiments, we explore the genetic network to identify genes whose expression correlates well with the high-glucose effect for fecundity. Some genes, such as *arf-1.1*, *fat-5*, *asm-3*, *lipl-1*, and *acdh-2*, did not revert their expression under high-glucose mixed-diet conditions, showing no correlation. However, other genes, such as *acs-2*, *fat-7*, *lipl-2*, *fil-1*, and *gba-4*, revert their expression levels, in part or fully, under the high-glucose mixed-diet conditions compared to those observed in OP (Supplementary Figure S4). Thus, the latter sets of genes are potential reporters reflecting dietary states under high-glucose conditions.

### Modulating PC levels through dietary supplementation of choline and methionine restored fecundity under high-glucose conditions

**FIGURE 6:**
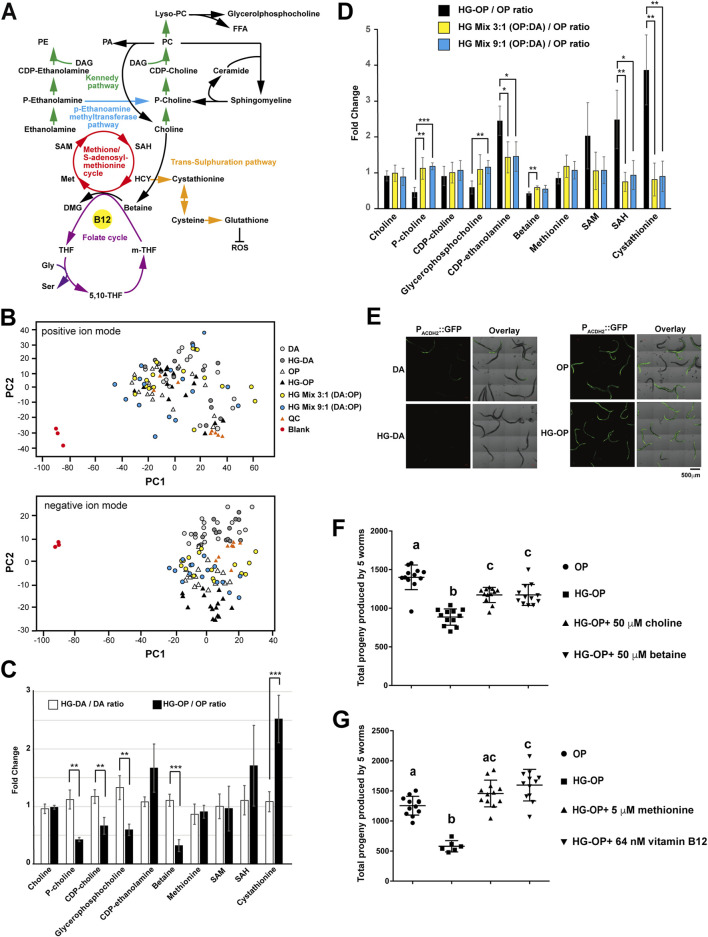
The choline–methionine metabolism can be modulated to revert fecundity decrease caused by high-glucose diets. **(A)** The metabolic pathways involved in PC synthesis are closely linked to choline metabolism and the methionine/S-adenosylmethionine (SAM) cycle that uses vitamin B12 as an essential cofactor. PA, phosphatidic acid; PE, phosphatidylethanolamine; DAG, diacylglycerol; FFA, free fatty acid, Lyso-PC, lysophosphatidylcholine; P-choline, phosphocholine; CDP-choline, cytidine 5′-diphosphocholine; P-ethanolamine, phosphoethanolamine; SAM, S-adenosylmethionine; SAH, S-adenosylhomocysteine; HCY, homocysteine; Met, methionine; DMG, dimethylglycine; m-THF, 5-methyltetrahydrofolate; THF, tetrahydrofolate; 5, 10-THF, 5, 10-methylenetetrahydrofolate; Gly, glycine, Ser, serine. **(B)** Aqueous-phase metabolites were isolated from *C. elegans* fed different diets and subjected to LC-MS analysis. The PCA plots, comparing difference among the six samples in both positive and negative ion modes, are shown. QC represents the mix of all six samples. **(C)** Aqueous-phase metabolites were isolated from *C. elegans* fed different diets and subjected to LC-MS analysis, followed by targeted searches to quantify the abundance of the metabolites shown. The data compare the metabolite ratios of OP diets under normal and high-glucose conditions with those of DA diets under the same conditions. Statistical analysis was performed using two-tailed Student’s *t*-test (**, *p* < 0.01; ***, *p* < 0.001; ns, not significant), and the data are presented as the mean ± s.d. n = 4. **(D)** Same as **(C)**, except that metabolite ratios in *C. elegans* fed the OP diet were compared with those in animals fed HG-OP, HG mix 3:1 (OP:DA), and HG mix 9:1 (OP:DA) diets. **(E)** The *C. elegans* strain VL714, harboring the B12 reporter *Pacdh-2*::GFP, was fed different diets and imaged using confocal microscopy. **(F)** *C. elegans* N2 strains were fed OP on NGM plates with (HG-OP) or without 100 mM glucose (OP), supplemented with 50 μM choline or 50 μM betaine. Reproductive output was determined, and data are presented as the mean+/−s.d. Statistical analysis was carried out using one-way ANOVA. **(G)** Same as F, except that 5 μM methionine or 64 nM vitamin B12 was supplemented.

We have demonstrated that a trend of reduced PC and increased TAG is closely correlated with a decrease in fecundity in *C. elegans* fed the HG-OP diet. In most cell types, including *C. elegans*, PC is synthesized via the Kennedy pathway (also known as the CDP-choline pathway), which uses exogenous choline from the environment to convert DAG to PC (Figure 6A). During choline metabolism, biosynthesis of phosphocholine, the first committed step in PC synthesis, can also be mediated through the action of phosphoethanolamine methyltransferases in *C. elegans*, which converts phosphoethanolamine into phosphocholine via three methylation steps, using SAM as the methyl donor (Figure 6A).

To analyze metabolism unambiguously, we performed the two-phase extraction method and profiled total aqueous-phase metabolites in *C. elegans* using MS-Dial, followed by multivariate analysis with PCA. The PCA plots serve as a reference for sample variation. As no distinct global metabolic differences were observed, it also suggests that worms fed different diets exhibit similar overall metabolic profiles (Figure 6B). However, targeted analysis revealed that although choline levels remained unchanged, several downstream metabolites of choline—including phosphocholine, cytidine 5′-diphosphocholine (CDP-choline), glycerophosphocholine, and betaine—were reduced in worms fed the HG-OP diet compared to those fed OP, coinciding with a reduction in PC observed in our lipidomic data (Figure 6C). In contrast, little to no change was observed in worms fed the HG-DA diet compared to those fed DA.

Intriguingly, we also found that animals fed HG-OP significantly elevated the level of cystathionine, a trans-sulfuration pathway intermediate metabolite downstream of the methionine cycle, and showed slightly higher CDP-ethanolamine and S-adenosyl-homocysteine (SAH) levels (Figures 6C,D). The changes in metabolites of choline and methionine/SAM pathways had led us to hypothesize that the choline–methionine/SAM metabolic axis may vary the dietary effect, thereby changing fecundity under high-glucose conditions. If true, we suspect the mixed diets that restore fecundity and overall lipid homeostasis, including the FA profile (Figure 4D) and PC and TAG abundance (Figures 5C,D), will also normalize levels of the choline and methionine/SAM metabolites under high-glucose conditions. Consistent with this hypothesis, we observed that, except for betaine, most choline and methionine/SAM metabolites returned to the normal OP diet level under the two mixed-diet conditions (Figure 6D).

The DA diet is rich in vitamin B12, a crucial cofactor for the production of SAM. Previous studies have shown that the *acdh-2* gene is induced several hundred-fold under B12-deficient conditions (MacNeil et al., 2013). Consistent with this, we observed strong *Pacdh-2::GFP* reporter signals in animals fed the OP diet, while only faint signals were detected in those fed DA (Figure 6E). Notably, this reporter showed no further changes when animals were exposed to high-glucose conditions on either diet. Similarly, qPCR analysis revealed no significant differences in *acdh-2* expression in animals fed HG-OP or mixed diets compared to those fed OP alone (Supplementary Figure S4). These results suggest that high-glucose conditions do not substantially alter B12 availability in animals.

To assess whether the choline–methionine/SAM metabolic axis is crucial for deciphering fecundity, we next performed supplementation experiments. Choline supplementation, by elevating PC synthesis via the Kennedy pathway, significantly, but not fully, improved total progeny number in worms fed the HG-OP diet (Figure 6F), supporting the idea that PC abundance is crucial for maintaining healthy fecundity. Intriguingly, betaine supplementation also significantly improved reproductive output in worms fed HG-OP. Although *C. elegans* lacks a gene coding for betaine–homocysteine methyltransferase, a recent report indicates that the *C. elegans* methionine synthase METR-1 can use both betaine and 5-methyltetrafolate as methyl donors to feed one-carbon metabolism (Kang et al., 2024). Thus, betaine may contribute to increased PC levels via the alternative phosphoethanolamine methyltransferase (PEMT) pathway by modulating methionine and SAM production. To directly assess the contribution of the PEMT pathway to improved fecundity, we supplemented methionine and vitamin B12, a cofactor for methionine synthase, in worms fed HG-OP. Remarkably, supplementing with either methionine or vitamin B12 fully restored the fecundity decrease observed in worms fed the HG-OP diet (Figure 6G). Given that dietary vitamin B12 activates SAM and PC synthesis (Han et al., 2024), these data support that high PC abundance is crucial for maintaining reproductive health. Altogether, these findings indicate that the choline–methionine metabolic axis plays a central role in mediating the dietary effects of DA and OP under high-glucose conditions, leading to differential impacts on fecundity.

### High glucose impaired RAS/ERK signaling in *C. elegans*, leading to reduced oocyte numbers

**FIGURE 7:**
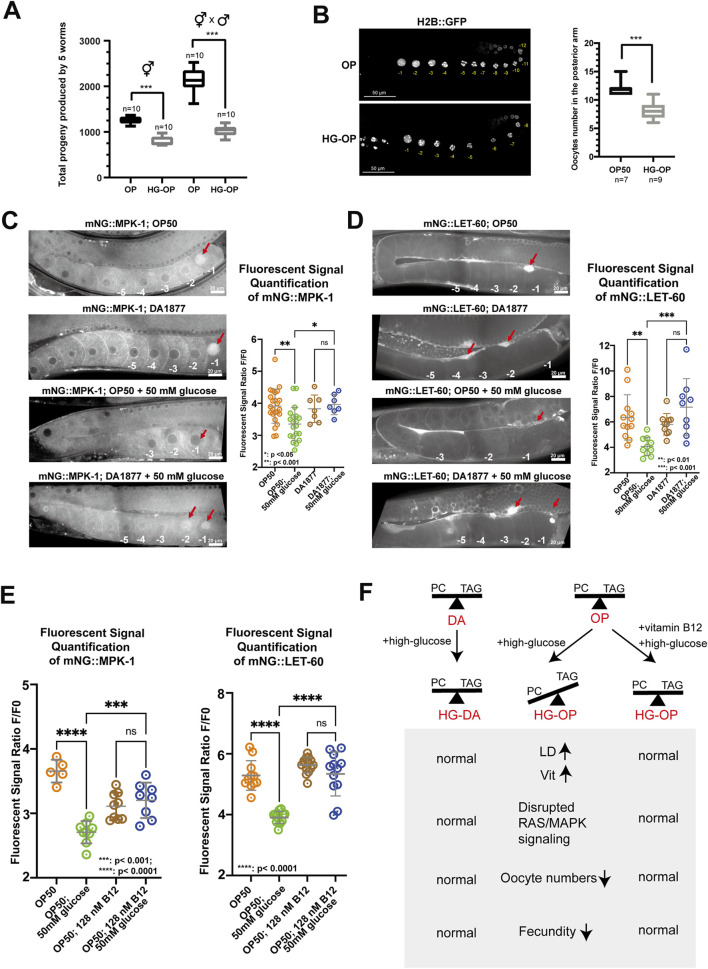
*C. elegans* fed HG-OP reduced oocyte numbers and showed altered oogenesis signaling. **(A)** *C. elegans* N2 strains, either hermaphrodites or hermaphrodites crossed with male individuals, were fed *E. coli* OP on NGM plates, without (OP) or with 100 mM glucose (HG-OP), to determine their progeny number. Reproductive output was determined, and data were plotted using Box and Whisker diagrams. Statistical analysis was performed using two-tailed Student’s *t*-test (***, *p* < 0.001). n = 10. **(B)** The *C. elegans* strain MAH-74, harboring GFP::H2B, was fed different diets and imaged using confocal microscopy. The total number of GFP::H2B foci in the cellularized oocytes in the posterior arm was quantified and plotted using Box and Whisker diagrams. Statistical analysis was performed using two-tailed Student’s *t*-test (***, *p* < 0.001; ns). **(C)** Representative images showing the germline expression of endogenously tagged mNG::MPK-1 under different dietary conditions. Red arrows highlight the enrichment of mNG::MPK-1 at the −1 oocytes during oocyte maturation. Quantification of mNG::MPK-1 fluorescent intensity at the −1 oocytes is shown on the right. Statistical analysis was performed using one-way ANOVA. **(D)** Representative images showing the expression of endogenously tagged mNG::LET-60 under different dietary conditions. Red arrows point to the enrichment of mNG::LET-60 in somatic sheath cells. Quantification of mNG::LET-60 fluorescent intensity in somatic sheath cells is shown on the right. Statistical analysis was performed using one-way ANOVA, with *p*-values indicated in the figure. **(E)** Quantification of mNG::MPK-1 and mNG::LET-60 fluorescent intensity at the −1 oocytes and somatic sheath cells, respectively, with dietary supplementation of vitamin B12. Statistical analysis was performed using one-way ANOVA, with *p*-values indicated in the figure. **(F)** Summary of the main findings of the study. The absence of vitamin B12 in OP diet limits the methionine/SAM cycle activity under high-glucose conditions, hindering efficient PC synthesis. Reduced PC levels compromise oogenesis signaling, contributing to reduced fecundity. In contrast, the HG-DA diet, rich in vitamin B12, supports healthy *C. elegans* fecundity by enabling vitamin B12 to maintain efficient PC synthesis, thereby exerting a protective effect against high glucose-induced fecundity decrease. Finally, B12 supplementation is sufficient to revert healthy fecundity in *C. elegans* fed the OP diet under high-glucose conditions.

We further explored the mechanistic impact of high glucose on fecundity in *C. elegans*. We observed a significant decrease in the reproductive output of both self-fertilized and male-mated hermaphrodite animals fed on the HG-OP diet, suggesting that high-glucose toxicity might impact female reproductive organs (Figure 7A). In *C. elegans* hermaphrodites, oogenesis begins at the distal end of the U-shaped gonad, where germline stem cells proliferate. As cells progress proximally, they undergo meiosis and mature into oocytes before fertilization occurs in the spermatheca. To characterize the potential high-glucose-induced impairment of gonad morphology and oogenesis, we imaged germline and oocytes labeled with the fluorescent reporters H2B::GFP (Figure 7B) and P*mex-5*::BFP (Supplementary Figure S5). By counting the cellularized oocytes at the proximal gonads, our data indicated that animals fed HG-OP had fewer oocytes per gonad than those fed the OP diet. In contrast, the DA diet increased the oocyte number relative to the OP diet, and the HG-DA diet did not exhibit a decrease in the oocyte count associated with high glucose addition (Supplementary Figure S5B).

Oogenesis in *C. elegans* is coordinated through signaling pathways among somatic sheath cells, the gonad, and male gametes. In adults, it occurs continuously in the presence of sperm and is regulated by receptor tyrosine kinase/RAS/ERK pathways, where changes in ERK signaling can alter oocyte numbers (Das et al., 2020; Lee et al., 2007). To examine RAS/ERK signaling under different diets, we first imaged the fluorescent ERK protein mNG::MPK-1. We found that endogenous mNG::MPK-1 was enriched in the −1 oocyte nucleus before fertilization under OP and DA diets (Figure 7C). However, the HG-OP diet reduced this enrichment, while the HG-DA diet maintained it and even enriched mNG::MPK-1 in the −2 oocyte nucleus. These data suggest that *C. elegans* fed HG-OP disrupts ERK signaling during oogenesis and oocyte maturation and that animals fed HG-DA may hyperactivate ERK signaling, potentially rescuing oocyte numbers under glucose toxicity.

Given that oocytes develop in close association with proximal gonadal sheath cells and are regulated by RAS-dependent signaling pathways (Greenstein, 2005; Li et al., 2012), we next examined whether high-glucose affects the endogenous expression of the RAS marker mNG::LET-60. Consistent with previous studies, we observed strong enrichment of mNG::LET-60 in the proximal gonadal sheath cells adjacent to the −1 oocyte of worms fed the OP and DA diets (Figure 7D), (Jayadev et al., 2023). However, this enrichment was markedly reduced in worms fed the HG-OP diet, while it was maintained in worms fed the HG-DA diet. These data suggest that high glucose disrupts extracellular RAS signaling during oogenesis and oocyte maturation, specifically in the context of the HG-OP diet. Finally, we investigated whether vitamin B12 supplementation to the HG-OP diet, which promotes SAM production for PC synthesis (Han et al., 2024), can rescue the disrupted signaling. mNG::LET-60 and mNG::MPK-1 expression patterns in the proximal gonad were restored to levels comparable to those in worms fed the OP diet alone (Figure 7E). Thus, these results are consistent with the notion that rewiring PC metabolism can mitigate glucose-induced oogenesis defects in *C. elegans*.

Altogether, we identified that high dietary glucose levels may exert deleterious effects by disrupting choline–methionine metabolic homeostasis (Figure 7F). Harmful high-glucose diets, such as *E. coli* OP50 for *C. elegans*, lead to a reduction in PC and an elevation of TAG, along with abnormalities in fat-carrying organelles such as LDs and yolks. These changes may further link to disrupted RAS/ERK signaling during oogenesis, ultimately leading to a decrease in fecundity. In contrast, protective high-glucose diets, such as *C. aquatica* DA1877 for *C. elegans*, help maintain PC and TAG homeostasis and normal RAS/ERK signaling during oogenesis under high-glucose conditions, thereby supporting healthy fecundity.

## Discussion

In this study, we investigate the interaction between *C. elegans* and bacterial diets to address high-glucose effects, which have been reported in various biological systems. Our analysis focused on two bacterial systems, resulting in distinct physiological outcomes for *C. elegans* reproduction under high-glucose conditions. Using this experimental paradigm, we applied omics analyses to investigate the specific mechanisms contributing to varying fecundity. Our findings provide compelling evidence linking variations in lipid homeostasis to the decrease in fecundity. Transcriptomic and lipidomic data revealed a number of changes in FAs and various lipid species in *C. elegans* fed high-glucose *E. coli* OP50. In contrast, these alterations were not observed in *C. elegans* fed the high-glucose *C. aquatica* DA1877 diet.

Lipidomic alterations may arise from lipid enzymes preferentially processing specific dietary components or from differentially regulated lipid biosynthesis pathways. Changes in FA and various lipid species may further affect membrane properties, such as fluidity and rigidity, and changes in membrane lipid species may subsequently damage protein and enzyme functions, disrupting normal animal physiology. To investigate the key mechanisms by which a high-glucose diet alters lipid homeostasis, we conducted a series of supplementation experiments to validate the link between lipids and fecundity. Although we observed a decrease in the MUFA FA18:1n7 and an increase in cyclopropane FA 19:0 in *C. elegan*s fed a high-glucose *E. coli* OP50 diet, supplementing FA18:1n7 did not seem to improve fecundity compared to treatments with other unsaturated FAs (Figure 4E). Studies have suggested that cyclopropane FAs, like unsaturated FAs, promote bilayer fluidity by interfering with lipid packing (Poger and Mark, 2015). Thus, it seems likely that a reduction in MUFAs, which are crucial for basic cellular functions, could potentially be compensated for by the elevation of cyclopropane FAs.

Through combined lipidomic and metabolomic analyses, we identified that the decrease in fecundity appears to be closely related to PC and TAG abundance, and the wiring of the choline–methionine cycle is likely the determinant. Genes regulating PC biosynthesis and the methionine/SAM cycle have been linked to fat storage and reproduction in *C. elegans*. For example, *sams-1*-deficient *C. elegans* have larger LDs, reduced body size, and lower progeny numbers (Li et al., 2011). Depletion of *pmt-1*, the gene for phosphoethanolamine methylation, reduces PC levels and increases LDs in the intestine (Li et al., 2011). The sphingomyelinase *asm-3* supports PC synthesis by enhancing the phosphocholine pool, and *asm-3* mutants display larger LDs in embryos, along with reproductive defects (Schmokel et al., 2016). Reciprocal regulation of PC and TAG synthesis is also observed in other biological systems. For example, PC treatment reduces TAG levels and benefits high-fat diet-induced obesity and fatty liver in mice (Lee et al., 2014), whereas disruption of PC synthesis increases TAG accumulation in the liver and intestinal cells (Lee and Ridgway, 2018). Thus, maintaining a proper balance between PC and TAG is essential for a healthy life.

Fecundity is a crucial trait influencing population dynamics. Our findings support the idea that high-glucose conditions may disrupt choline–methionine metabolic wiring in *C. elegans* fed OP, leading to reduced PC production, and, consequently, compromised reproductive output. The restoration of fecundity by methionine and vitamin B12 supplementation suggests that the synthesis or utilization of SAM may be impaired in worms fed the HG-OP diet, contributing to reduced PC levels. Supporting this notion, our microarray data show the activation of a B12-responsive transcriptional program under low SAM conditions in HG-OP-fed worms, as evidenced by increased expression of *pmp-5*, *msra-1*, and *nhr-114*, alongside repression of *cbs-1* (Giese et al., 2020). In contrast, *C. elegans* fed the DA diet, rich in vitamin B12, exhibited enhanced SAM production. Notably, high-glucose-induced *sams-1* expression in DA-fed worms aligns with the hypothesis that increased SAM-driven PC synthesis helps protect against glucose toxicity (Supplementary Figure S6). In addition to its B12 content, DA also harbors endogenous genes for PC synthesis. Thus, it is plausible that enhanced choline availability represents another possible mechanism by which DA confers protection under high-glucose conditions. Although the precise mechanism by which high glucose disrupts the choline–methionine pathway remains unclear, it is worth investigating whether glucose-induced glycation and/or lipid peroxidation may directly or indirectly impair enzymes in the choline–methionine pathway. Furthermore, it may be interesting to determine whether dietary components unique to the DA strain may contribute additional protective effects against such disruptions. Comparative metabolomic profiling of DA and OP will be instrumental in revealing additional insights.

In *C. elegans*, oocyte quality and quantity are tightly regulated by the oncogenic RAS/ERK signaling pathways (Arur et al., 2011; Arur et al., 2009; Das et al., 2020; Lee et al., 2007). Our data indicate that high glucose significantly suppresses the expression of RAS/ERK reporters in both germline and somatic sheath cells in *C. elegans* fed OP (Figure 7). Although we cannot completely rule out reduced oogenesis signaling as an indirect effect of reduced oocyte production, our data link PC reduction to compromised oogenesis. Interestingly, vitamin B12 supplementation appeared to hyperactivate RAS/ERK expression, restoring fecundity that was otherwise reduced by high glucose in *C. elegans* fed OP (Figure 7E). This finding aligns with prior studies suggesting that hyperactivation of RAS/ERK pathways may require PC biosynthesis, which is essential for cell survival under excitotoxic stress and germline or vulva cell fate decisions (Crook et al., 2016; Laranjeira et al., 2024). PC is critical for cellular structure and function, particularly in membrane integrity and signaling. Under high-glucose conditions, reduced PC levels may disrupt membrane properties, affecting key processes such as endocytic recycling and vesicular transport. Additionally, PC depletion may alter membrane domain organization, impairing signaling pathways essential for oogenesis. The concomitant formation of larger LDs in oocytes with reduced PC could further hinder lipid mobilization, limiting energy availability during oocyte development. The exact mechanism by which PC influences fecundity and reproductive health awaits further investigation to clarify.

Altogether, our study underscores the critical role of PC metabolism in preserving reproductive health and offers insights into potential dietary strategies to counteract high glucose-induced reproductive defects. Further investigation is warranted to elucidate the direct impact of excess glucose on the choline–methionine metabolic axis and determine how PC functions during oogenesis under conditions of glucose toxicity and vitamin B12 supplementation. These insights may also deepen our understanding of the molecular pathways linking nutrient stress—particularly glucose overload—to carcinogenic signaling and oncogenic transformation.

## Data Availability

The raw data are available upon request from the corresponding author.
