# Supplementary material for: High-glucose diets differentially modulate phosphatidylcholine metabolism and fecundity in Caenorhabditis elegans
Source: Front Cell Dev Biol. 2025 Aug 29;13:1622695. doi: 10.3389/fcell.2025.1622695 (PMC12425989; doi:10.3389/fcell.2025.1622695)
Supplement: Supplementary file 1 [file DataSheet2.pdf]

Figure S2

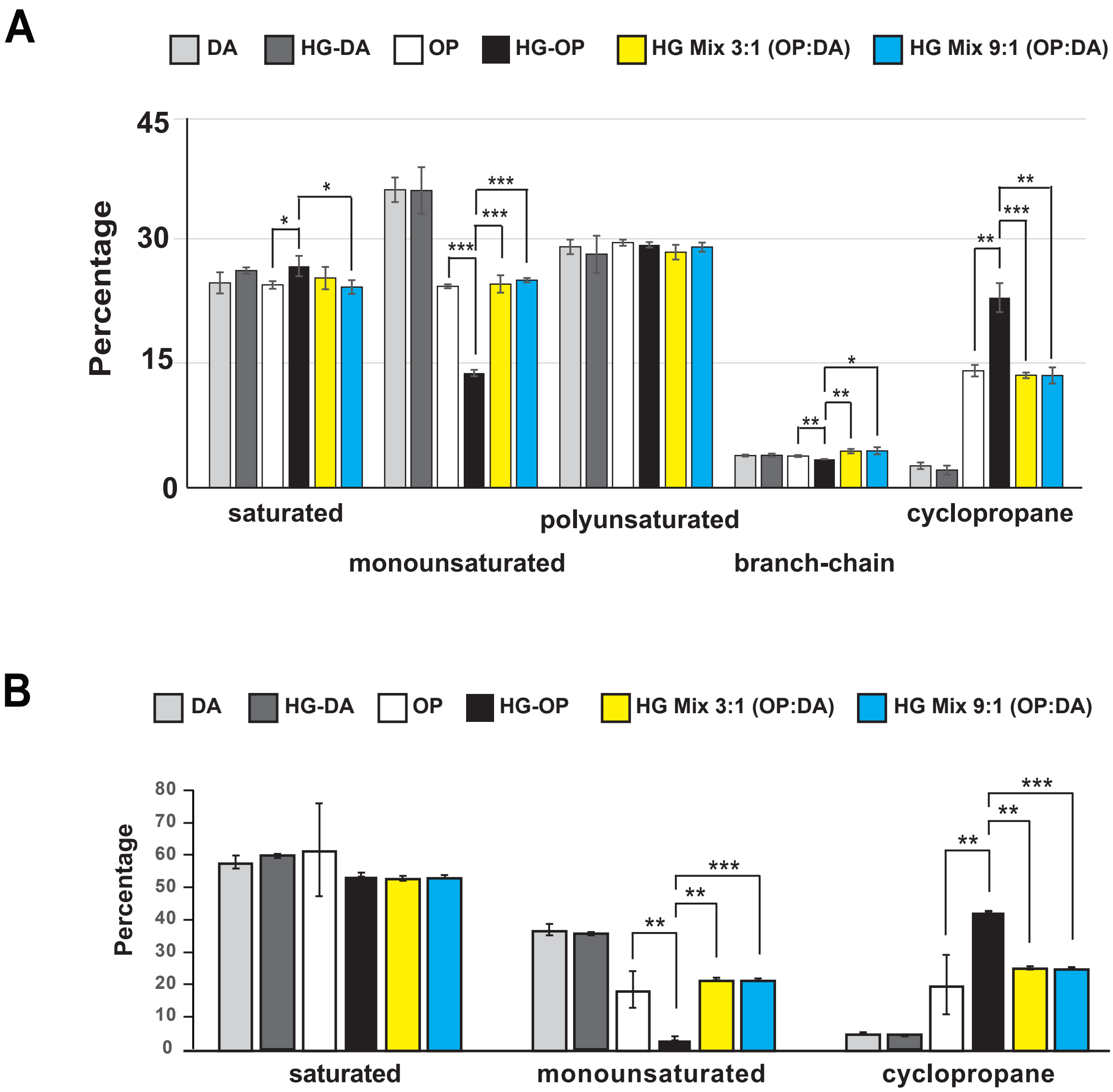

Figure S2: Mixing a small portion of DA into OP significantly alters FA profiles under high-glucose conditions (A) Lipids were extracted from *C. elegans* fed different dietary bacteria (A) or from dietary bacteria (B), converted to FA methyl esters, and analyzed by gas-chromatography-mass spectrometry (GC-MS).The FA species were grouped into saturated, monounsaturated, branch-chain or cyclopropane FAs, and dada was presented as mean+/-s.d. *, P<0.05; **, P<0.01; ***, P<0.001.
