# Supplementary material for: High-glucose diets differentially modulate phosphatidylcholine metabolism and fecundity in Caenorhabditis elegans
Source: Front Cell Dev Biol. 2025 Aug 29;13:1622695. doi: 10.3389/fcell.2025.1622695 (PMC12425989; doi:10.3389/fcell.2025.1622695)
Supplement: Supplementary file 2 [file DataSheet4.pdf]

Figure S4

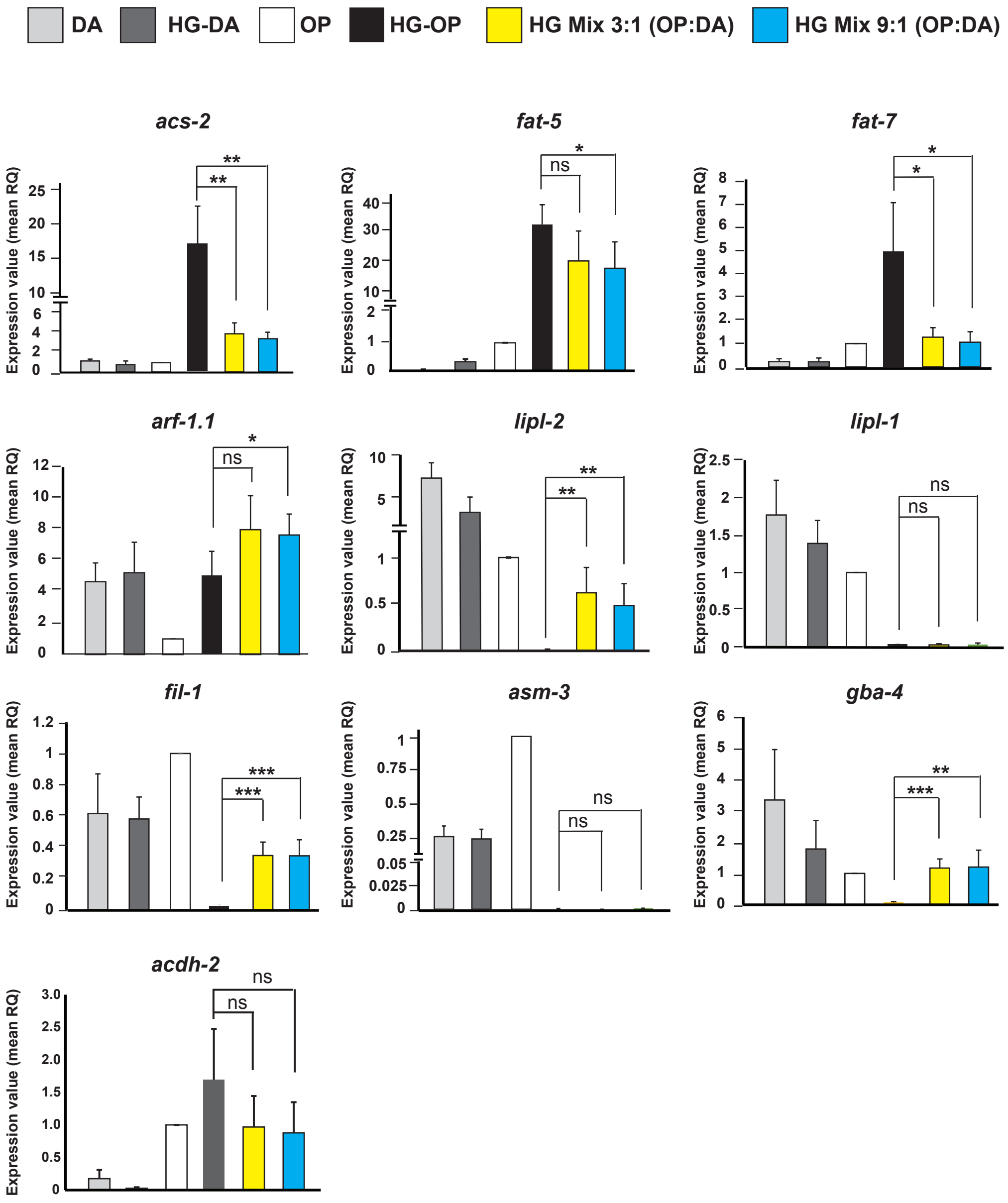

Figure S4: ***C. elegans*** **fed DA, HG-DA, OP, HG-OP, and two high-glucose mix diets, HG Mix 3:1(OP:DA) and HG Mix 9:1 (OP:DA), were grown from L1 to gravid young adult stage. qPCR analysis comparing RNA abundance of indicated genes in various dietary conditions.** The RNA abundance of OP is set as 1. Statistics is done using two-tailed Student t test (*, $p < 0.05$; **, $p < 0.01$; ***, $p < 0.001$; ns, not significant) and the data are presented as mean RQ+/-s.d. n=4.
