# Supplementary material for: High-glucose diets differentially modulate phosphatidylcholine metabolism and fecundity in Caenorhabditis elegans
Source: Front Cell Dev Biol. 2025 Aug 29;13:1622695. doi: 10.3389/fcell.2025.1622695 (PMC12425989; doi:10.3389/fcell.2025.1622695)
Supplement: Supplementary file 3 [file DataSheet6.pdf]

Figure S6

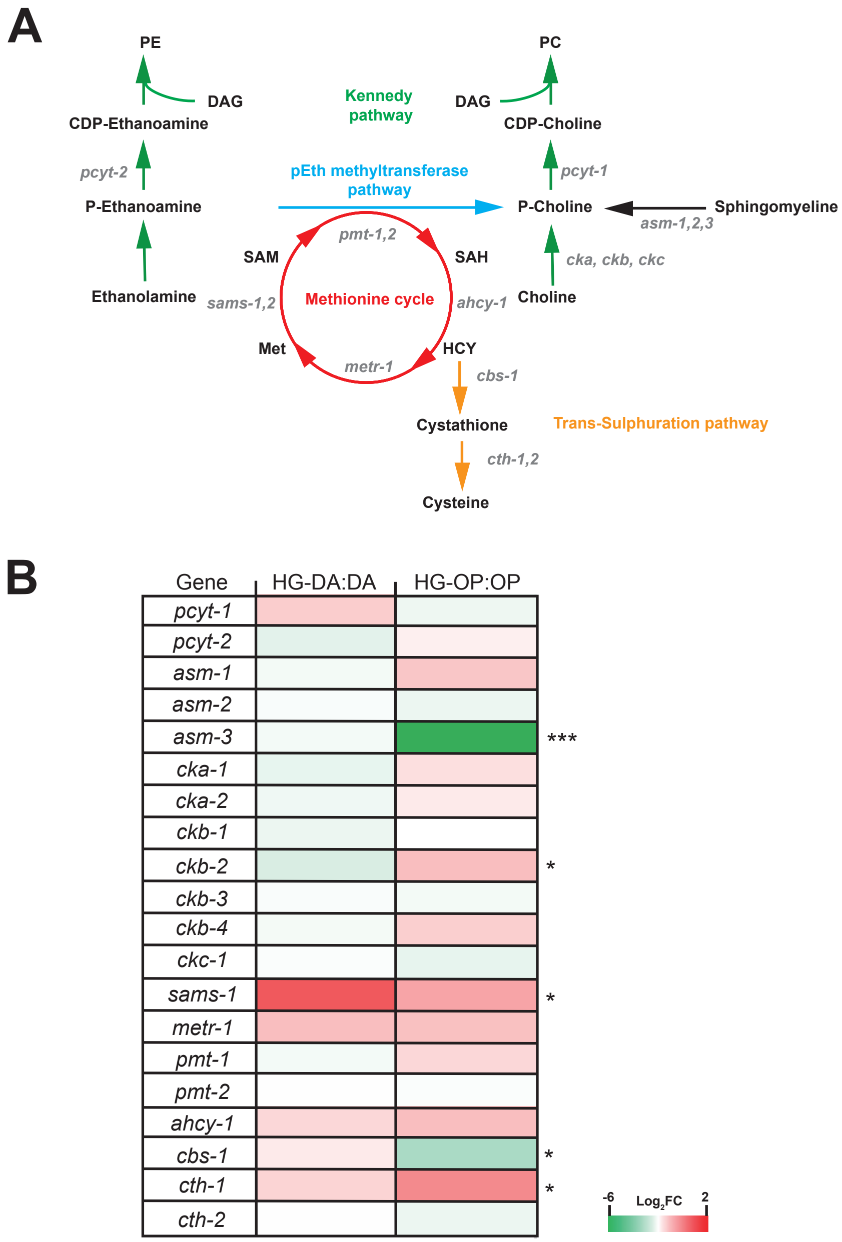

Figure S6: **Regulation of choline and methionine metabolism-related genes in *C. elegans* under high-glucose dietary conditions.**(A)The Kennedy pathway (green), the phosphoethanolamine (pEh) methyltransferase pathway (blue), the methionine/SAM cycle (red), and the trans-sulphuration pathway (orange). Genes associated with each metabolic step are shown in grey. PE, phosphatidylethanolamine; DAG, diacylglycerol; P-choline, phosphocholine; CDP-choline, Cytidine 5'-diphosphocholine; P-ethanolamine, phosphoethanolamine; CDP-ethanolamine, Cytidine 5'-diphosphoethanolamine; SAM, S-adenosylmethionine; SAH, S-adenosylhomocysteine; HCY, homocysteine; Met, methionine. (B) *C. elegans* fed DA, HG-DA, OP, HG-OP were harvested and the isolated mRNA from animals were subjected for microarray analysis. Gene expression ratio was shown as heat map. P-value comparing HG-OP:OP ratio to HG-DA:DA ratio are indicated (*, $p < 0.05$; **, $p < 0.01$; ***, $p < 0.001$). Data represents three independent experiments.
