# Supplementary material for: High-glucose diets differentially modulate phosphatidylcholine metabolism and fecundity in Caenorhabditis elegans
Source: Front Cell Dev Biol. 2025 Aug 29;13:1622695. doi: 10.3389/fcell.2025.1622695 (PMC12425989; doi:10.3389/fcell.2025.1622695)
Supplement: Supplementary file 4 [file DataSheet3.pdf]

Figure S3_1

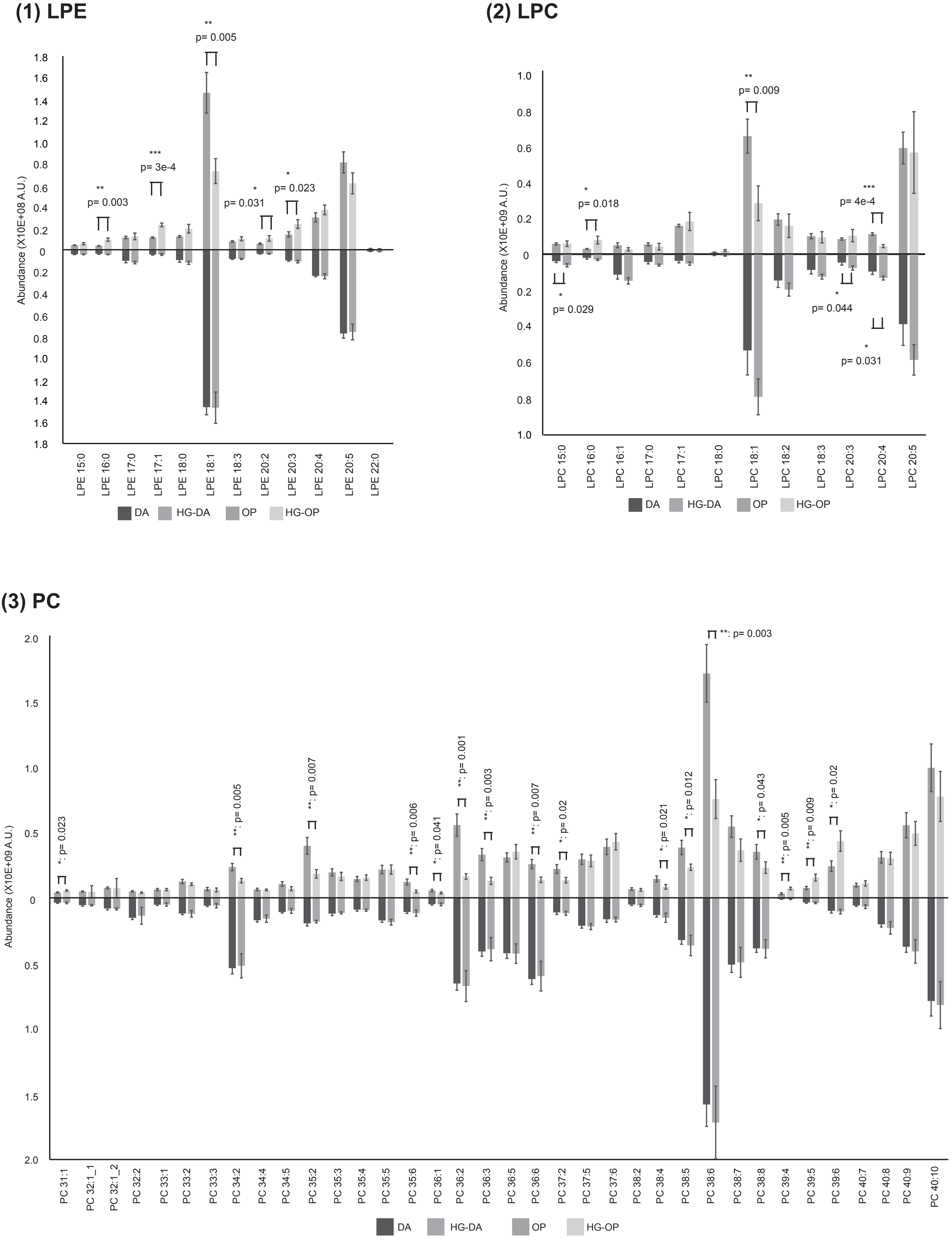

(1) LPE
Abundance (X10E+08 A.U.)
** p= 0.003
*** p= 3e-4
** p= 0.005
p= 0.031
* p= 0.023
LPE 15:0
LPE 16:0
LPE 17:0
LPE 17:1
LPE 18:0
LPE 18:1
LPE 18:3
LPE 20:2
LPE 20:3
LPE 20:4
LPE 20:5
LPE 22:0
DA
HG-DA
OP
HG-OP
(2) LPC
Abundance (X10E+09 A.U.)
* p= 0.018
* p= 0.029
** p= 0.009
*** p= 4e-4
* p= 0.044
* p= 0.031
LPC 15:0
LPC 16:0
LPC 16:1
LPC 17:0
LPC 17:1
LPC 18:0
LPC 18:1
LPC 18:2
LPC 18:3
LPC 20:3
LPC 20:4
LPC 20:5
DA
HG-DA
OP
HG-OP
(3) PC
Abundance (X10E+09 A.U.)
*: p= 0.023
**: p= 0.005
**: p= 0.007
**: p= 0.006
*: p= 0.041
**: p= 0.001
**: p= 0.003
**: p= 0.007
*: p= 0.02
*: p= 0.021
*: p= 0.012
**: p= 0.003
*: p= 0.043
**: p= 0.005
**: p= 0.009
*: p= 0.02
PC 31:1
PC 32:1_1
PC 32:1_2
PC 32:2
PC 33:1
PC 33:2
PC 33:3
PC 34:2
PC 34:4
PC 34:5
PC 35:2
PC 35:3
PC 35:4
PC 35:5
PC 35:6
PC 36:1
PC 36:2
PC 36:3
PC 36:5
PC 36:6
PC 37:2
PC 37:5
PC 37:6
PC 38:2
PC 38:4
PC 38:5
PC 38:6
PC 38:7
PC 38:8
PC 39:4
PC 39:5
PC 39:6
PC 40:7
PC 40:8
PC 40:9
PC 40:10
DA
HG-DA
OP
HG-OP

Figure S3_2

(4) PS

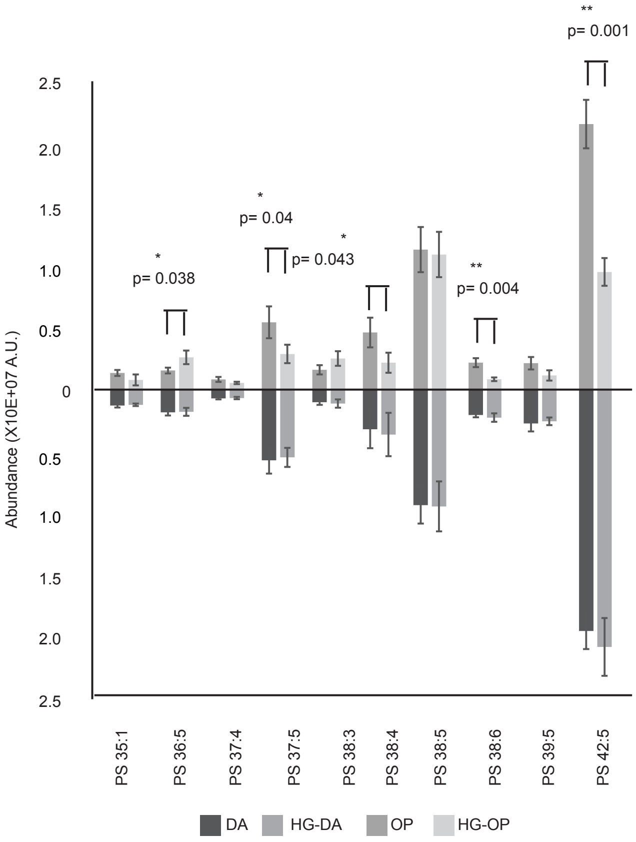

(5) PG

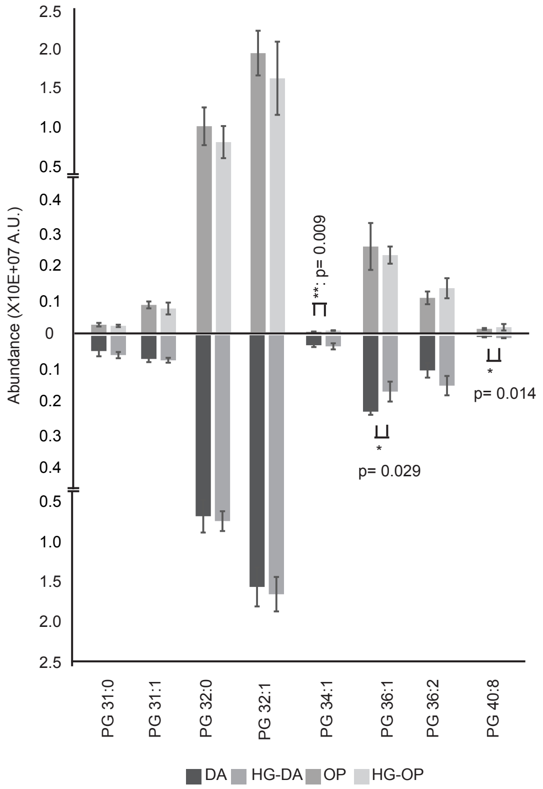

(6) PA

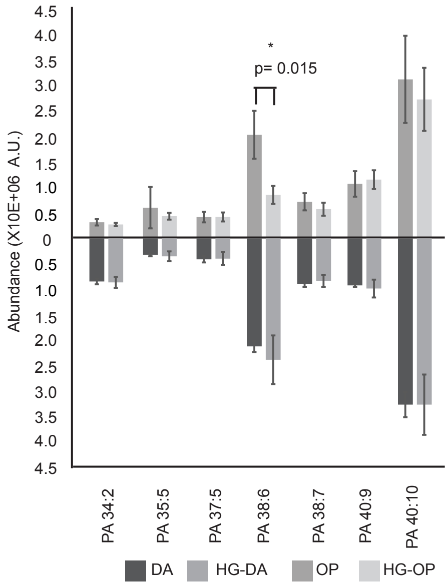

(7) PI

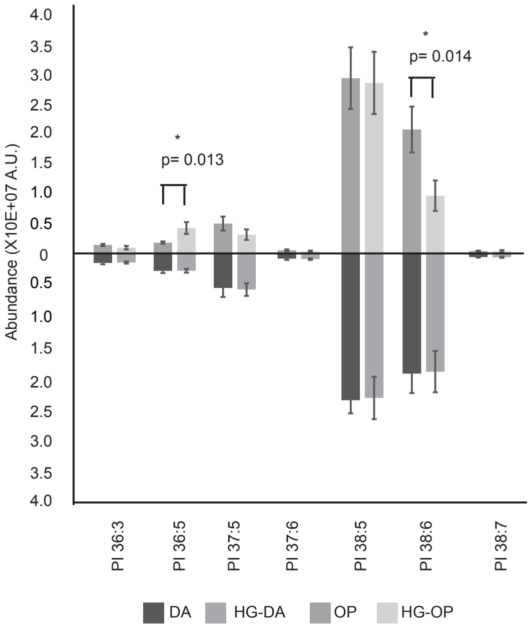

(8) DAG

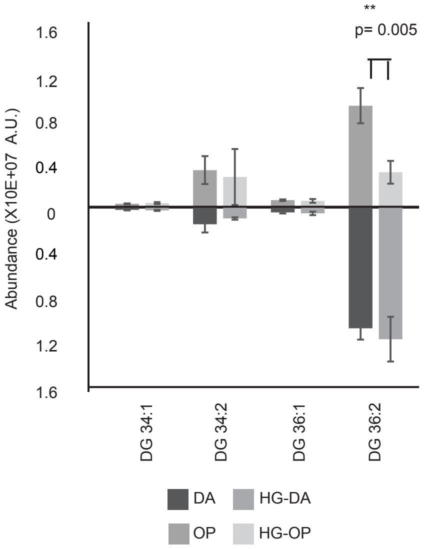

Figure S3_3

(9) PE

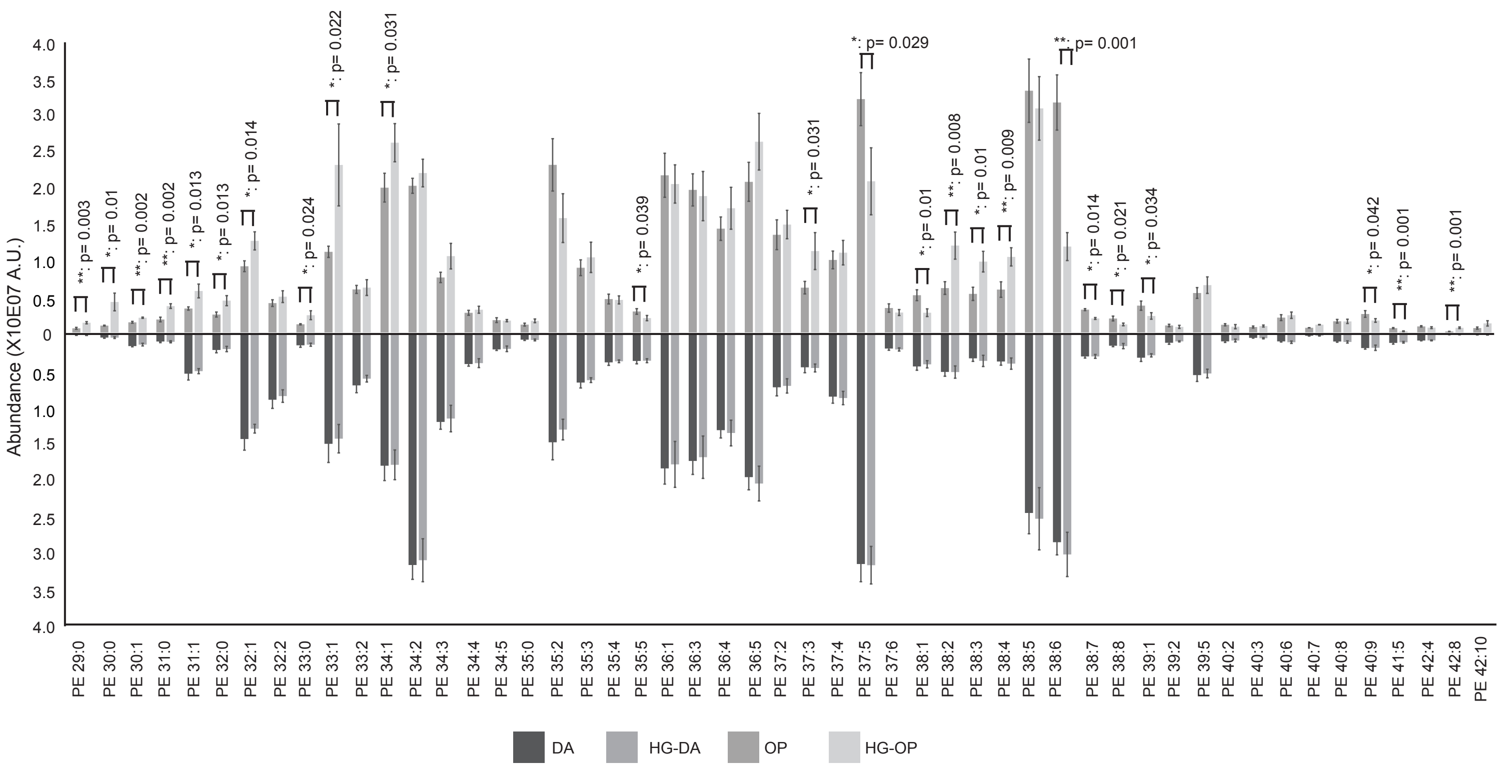

(10) TAG

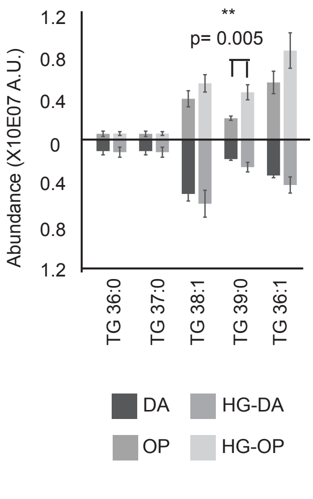

(11) TAG

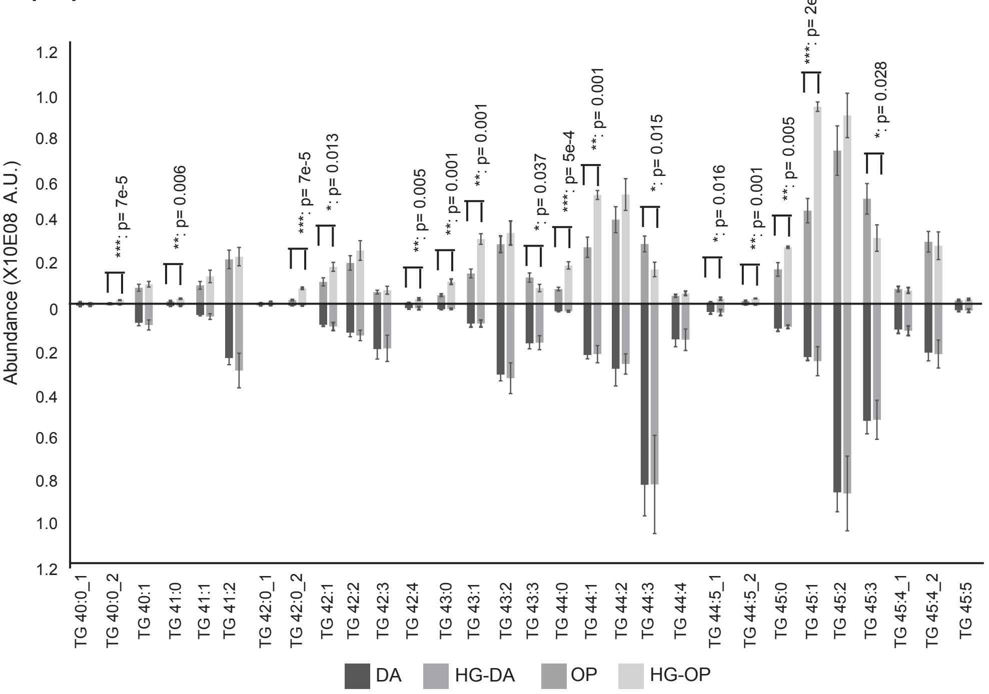

Figure S3_4

(12) TAG

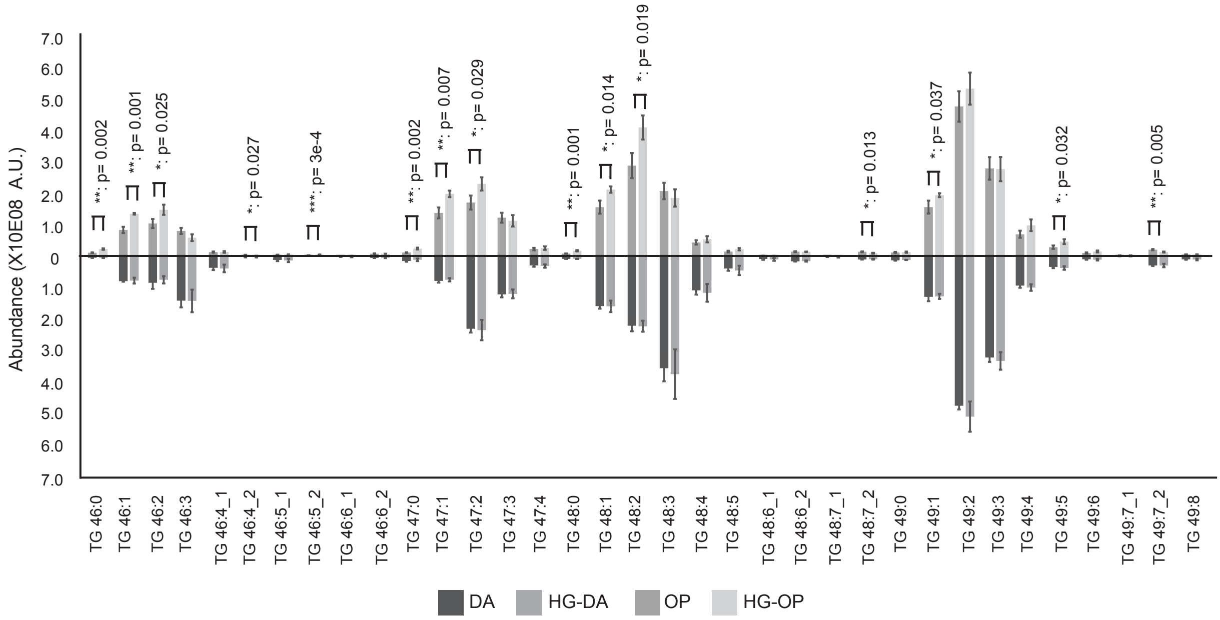

(13) TAG

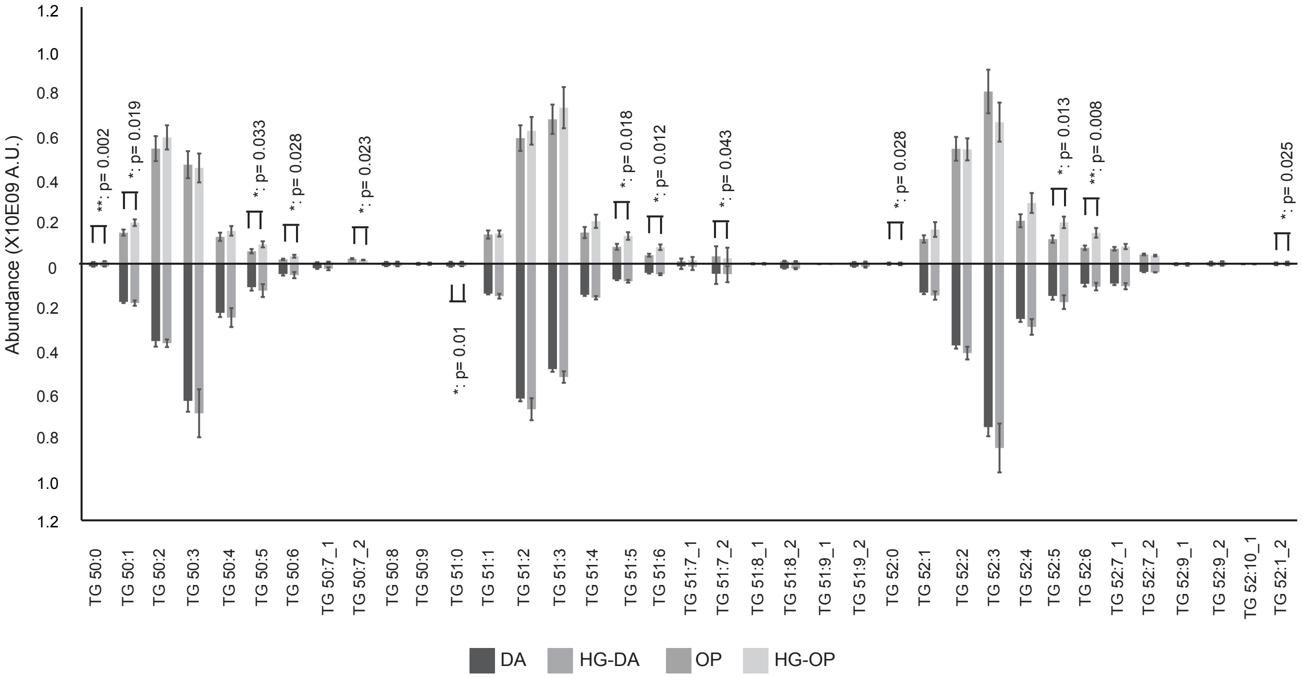

(14) TAG

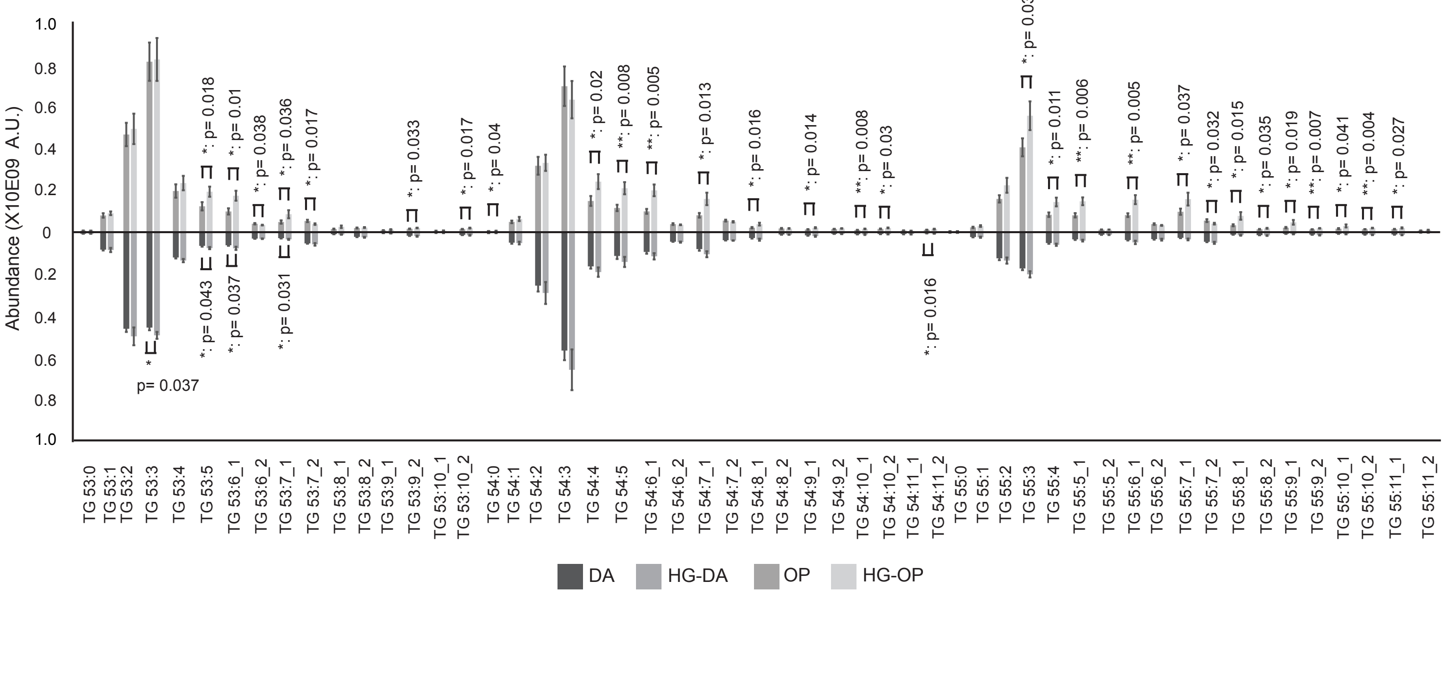

Figure S3_5

(15) TAG

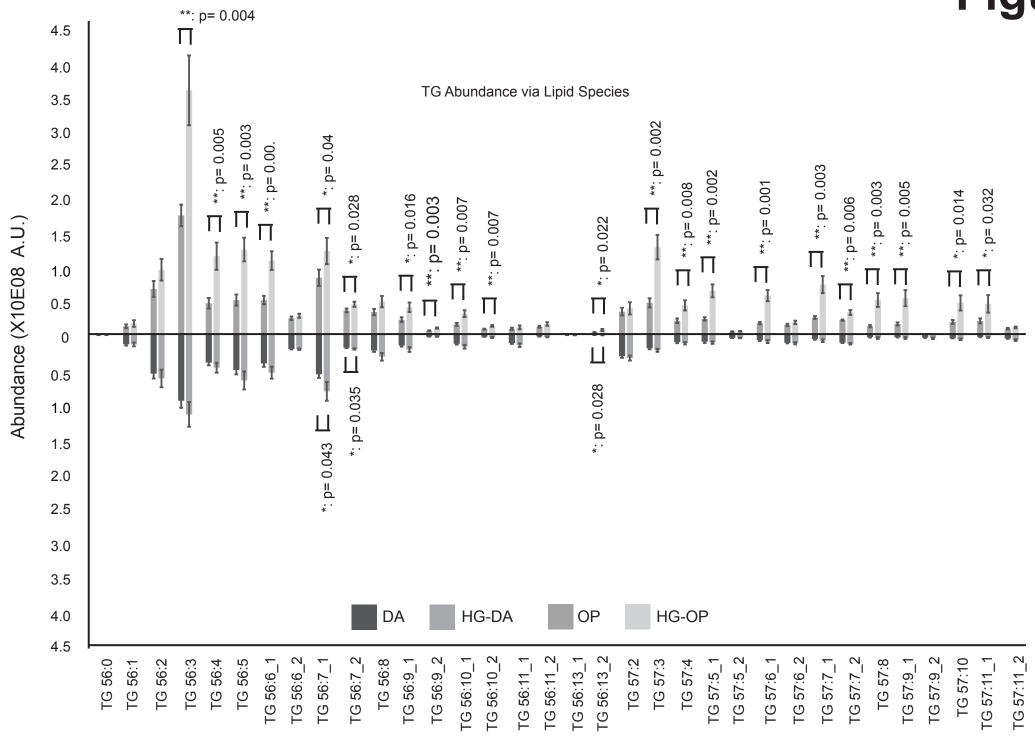

(16) TAG

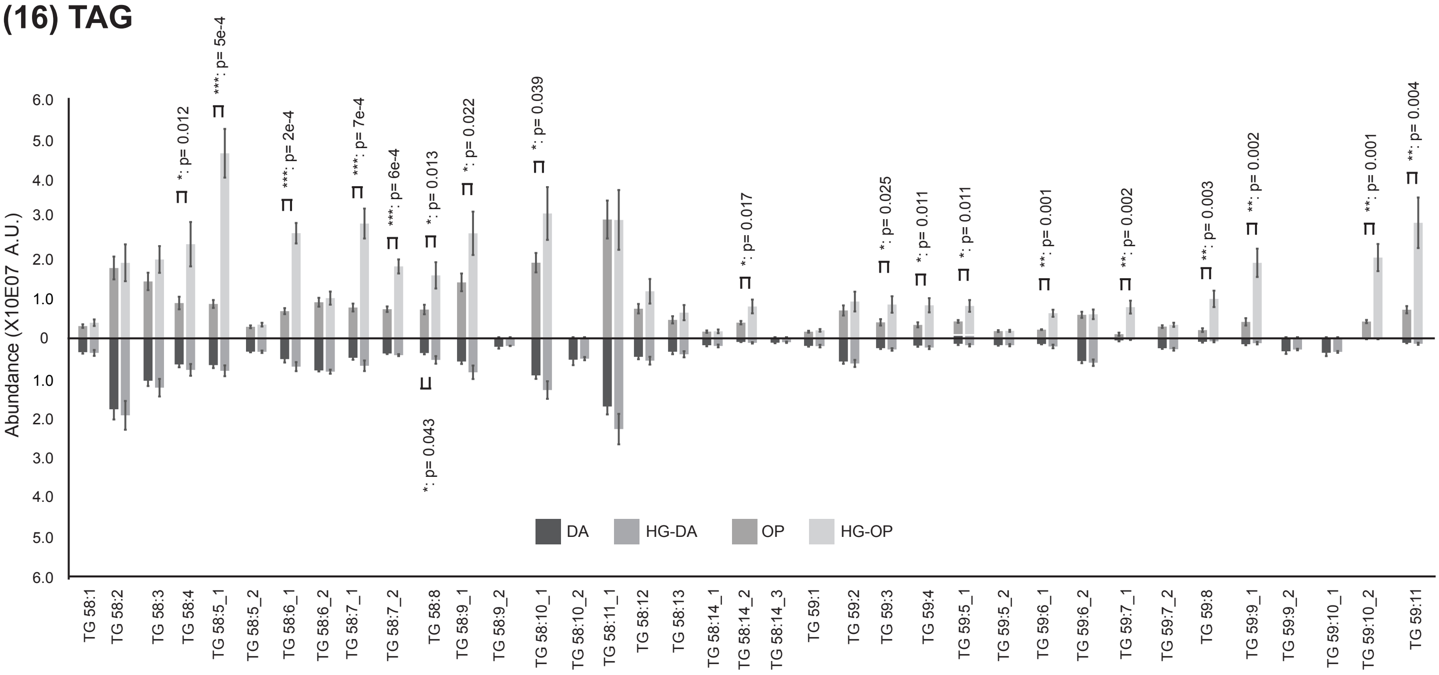

(17) TAG

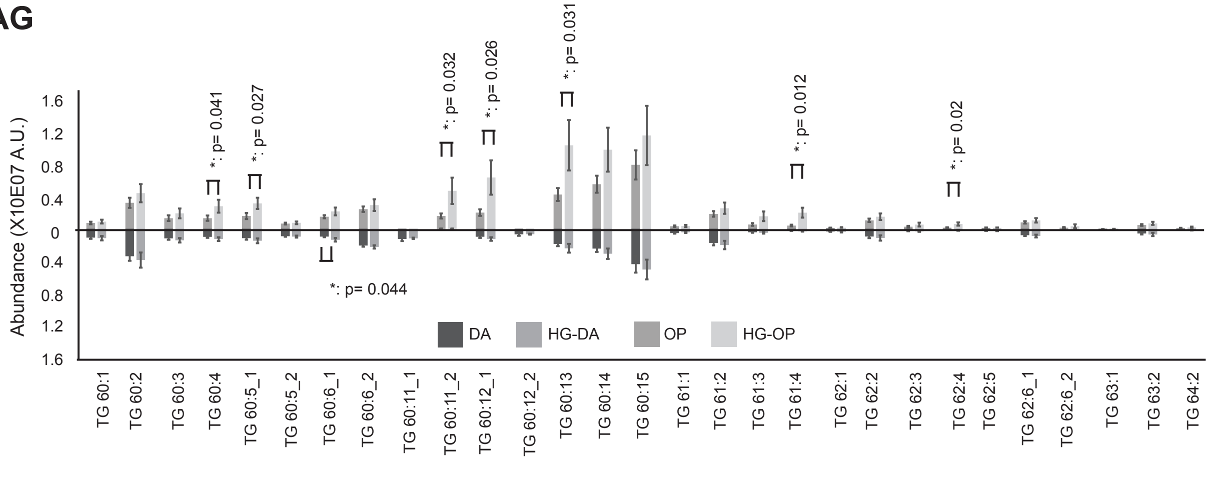

Figure S3: Lipidomic analyses identified various changes on lipid species specially associated with HG-OP
Lipids were extracted from *C. elegans* fed different diets and analyzed by liquid-chromatography-mass spectrometry (LC-MS). The species within each type of glycerolipid were compared. Data was presented as mean+/-s.d.
