# Supplementary figures and images for: High-glucose diets differentially modulate phosphatidylcholine metabolism and fecundity in Caenorhabditis elegans
Source: Front Cell Dev Biol. 2025 Aug 29;13:1622695. doi: 10.3389/fcell.2025.1622695 (PMC12425989; doi:10.3389/fcell.2025.1622695)

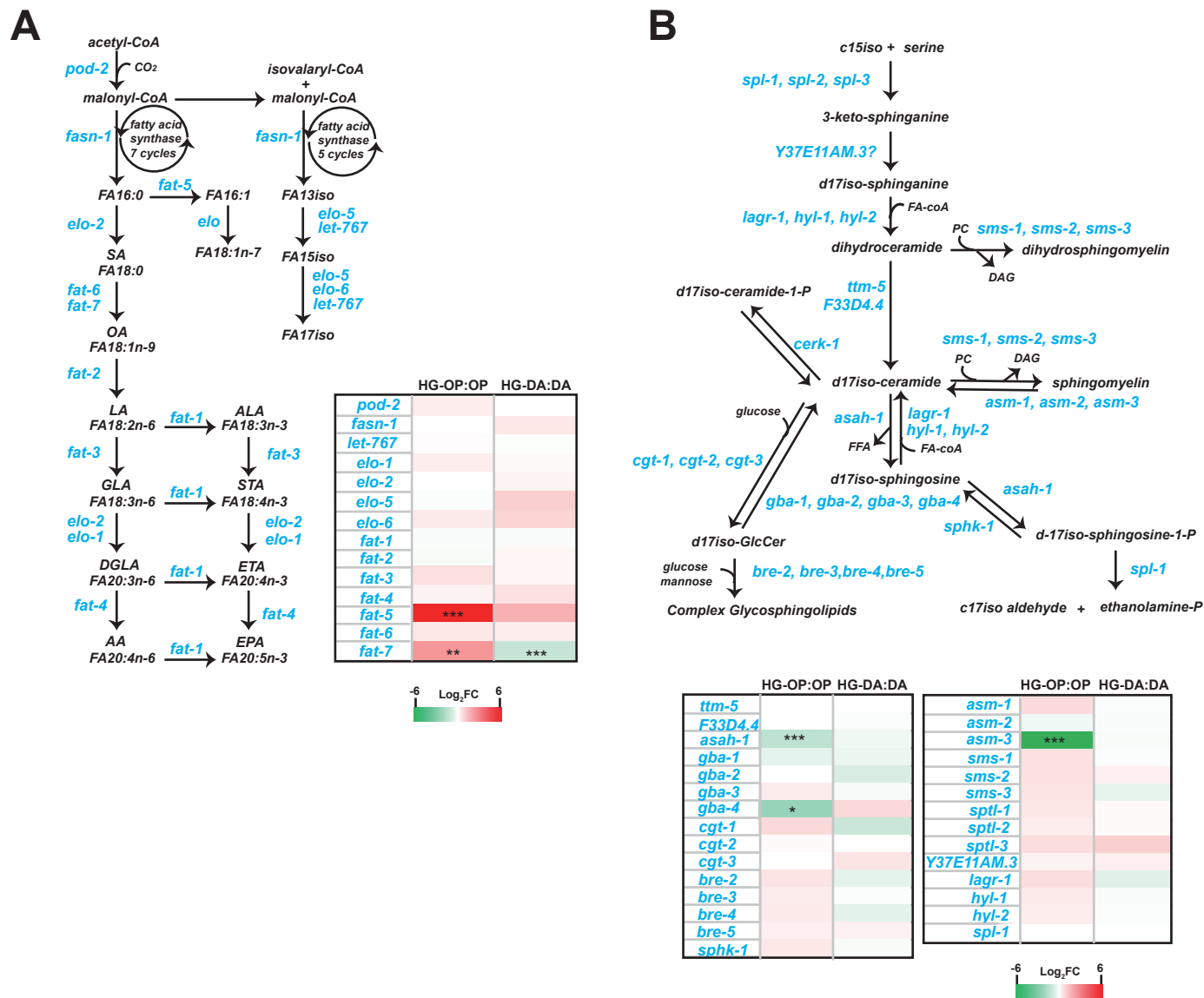

Supplement: Supplementary file 5 [file DataSheet1.pdf]
