# Supplementary material for: High-glucose diets differentially modulate phosphatidylcholine metabolism and fecundity in Caenorhabditis elegans
Source: Front Cell Dev Biol. 2025 Aug 29;13:1622695. doi: 10.3389/fcell.2025.1622695 (PMC12425989; doi:10.3389/fcell.2025.1622695)
Supplement: Supplementary file 6 [file DataSheet5.pdf]

Figure S5

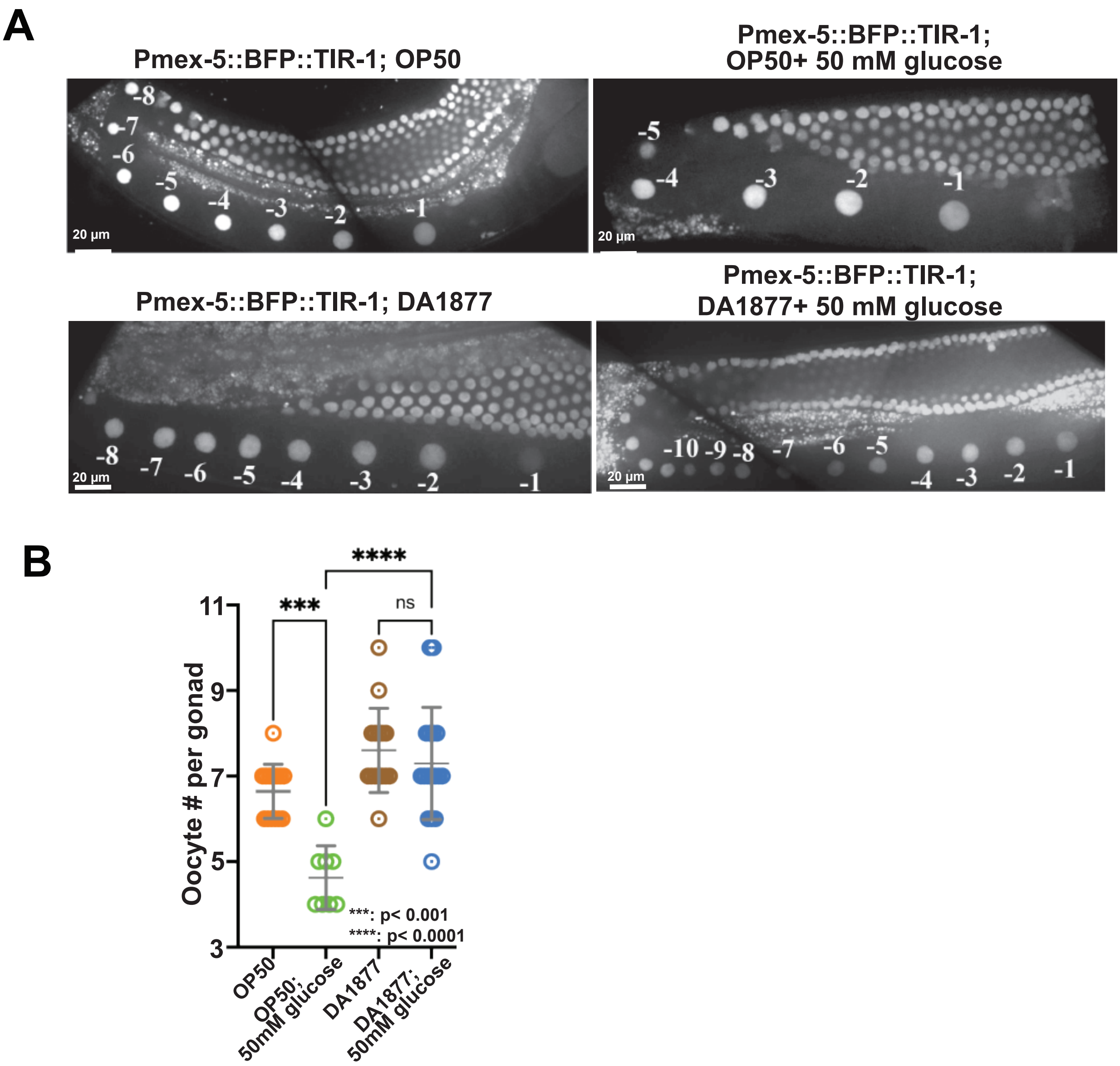

Figure S5: ***C. elegans* fed the HG-OP diet had fewer oocytes per gonad compared to those fed the OP diet. In contrast, *C. elegans* fed the HG-DA diet did not reduce oocyte numbers relative to those fed the DA diet.** (A) Representative images showing the germline expression of nuclei marker Pmex-5::BFP under varying dietary conditions. (B) Quantification of the oocyte numbers per gonad under various conditions. Statistical analysis was performed using one-way ANOVA.
